# Supplementary material for: Skyrmion dynamics in a frustrated ferromagnetic film and current-induced helicity locking-unlocking transition
Source: Nat Commun. 2017 Nov 23;8:1717. doi: 10.1038/s41467-017-01785-w (PMC5700181; doi:10.1038/s41467-017-01785-w)
Supplement: Supplementary file 1 — Supplementary Information [file 41467_2017_1785_MOESM1_ESM.pdf]

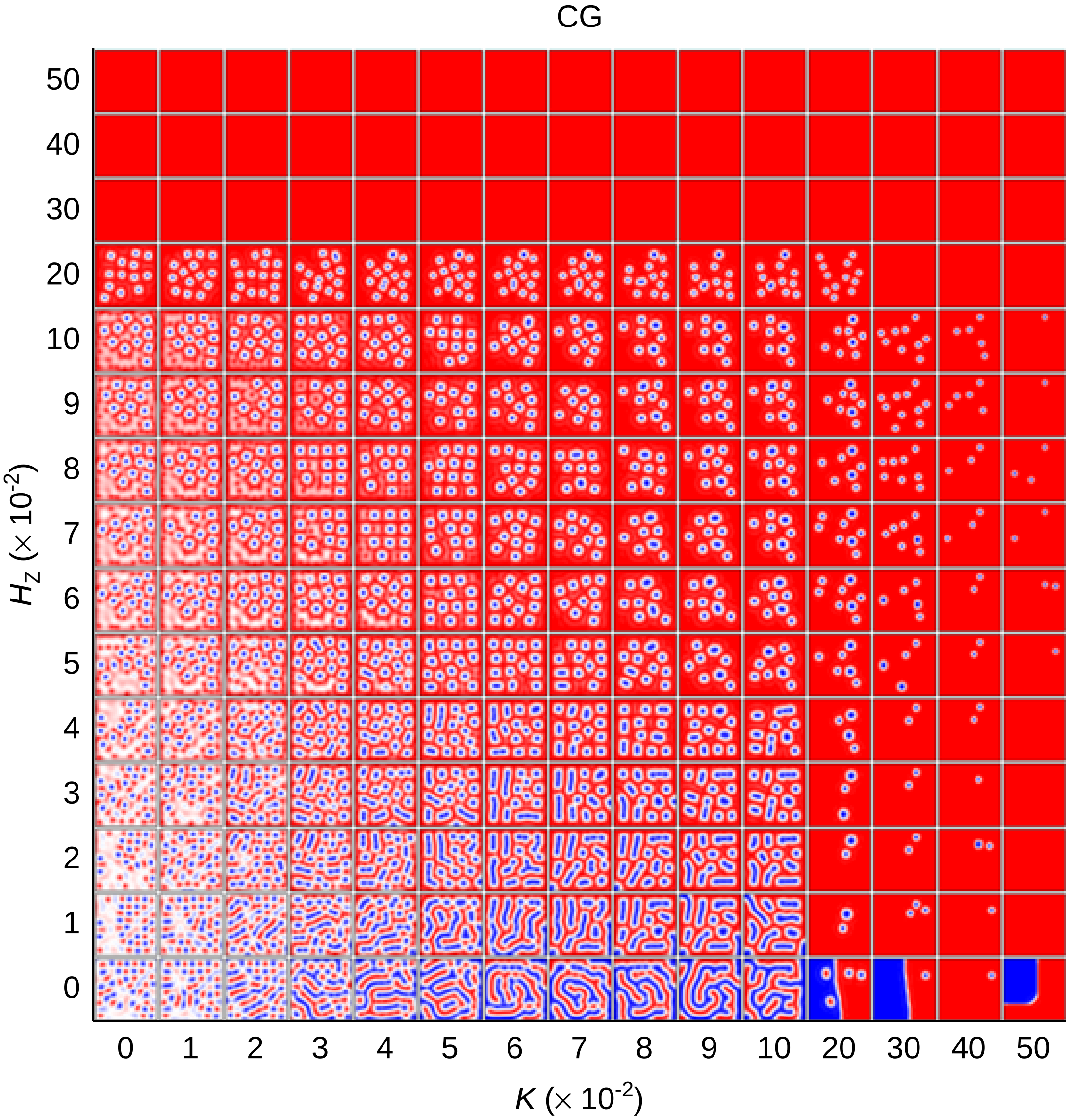

Supplementary Figure 1. **Typical metastable states (as functions of K and H_z) obtained by relaxing the magnetic thin film with a random initial spin configuration.** The out-of-plane spin component is color-coded: blue is into the plane, red is out of the plane, white is in-plane. The model is a square element (40×40 spins) with the OBC. The fixed parameters (in units of $J_1 = 1$) are $J_2 = -0.8$ and $J_3 = -1.2$. K and H_z are varied between $0 \sim 0.5$, respectively. The simulation is carried out by the OOMMF CG minimizer.

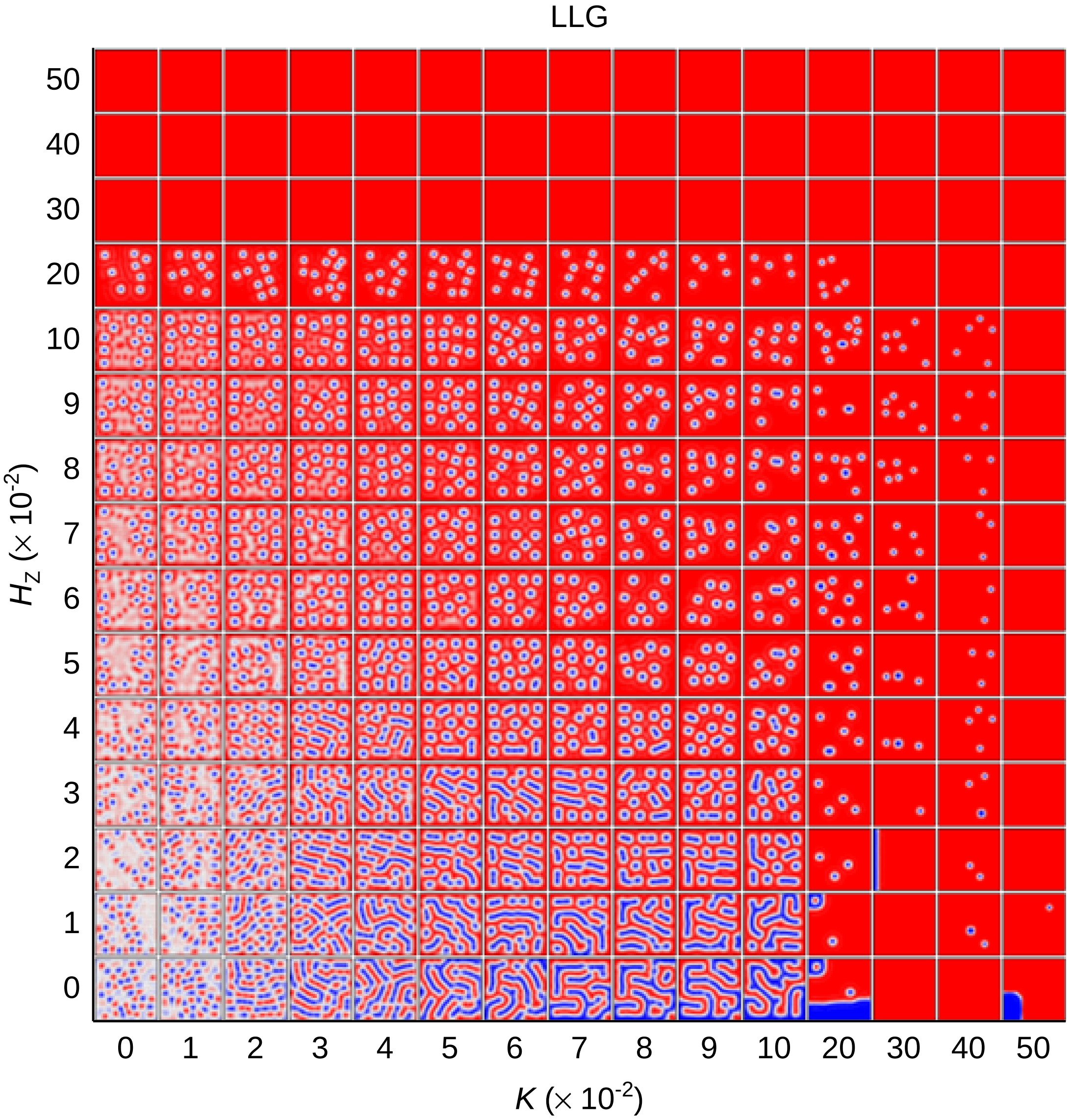

Supplementary Figure 2. **Typical metastable states (as functions of K and H_z) obtained by relaxing the magnetic thin film with a random initial spin configuration.** The out-of-plane spin component is color-coded: blue is into the plane, red is out of the plane, white is in-plane. The model is a square element (40×40 spins) with the OBC. The fixed parameters (in units of $J_1 = 1$) are $J_2 = -0.8$ and $J_3 = -1.2$. K and H_z are varied between $0 \sim 0.5$, respectively. The simulation is carried out by the OOMMF solver integrating the LLG equation. The results shown in Supplementary Figs. 1 and 2 are qualitatively similar.

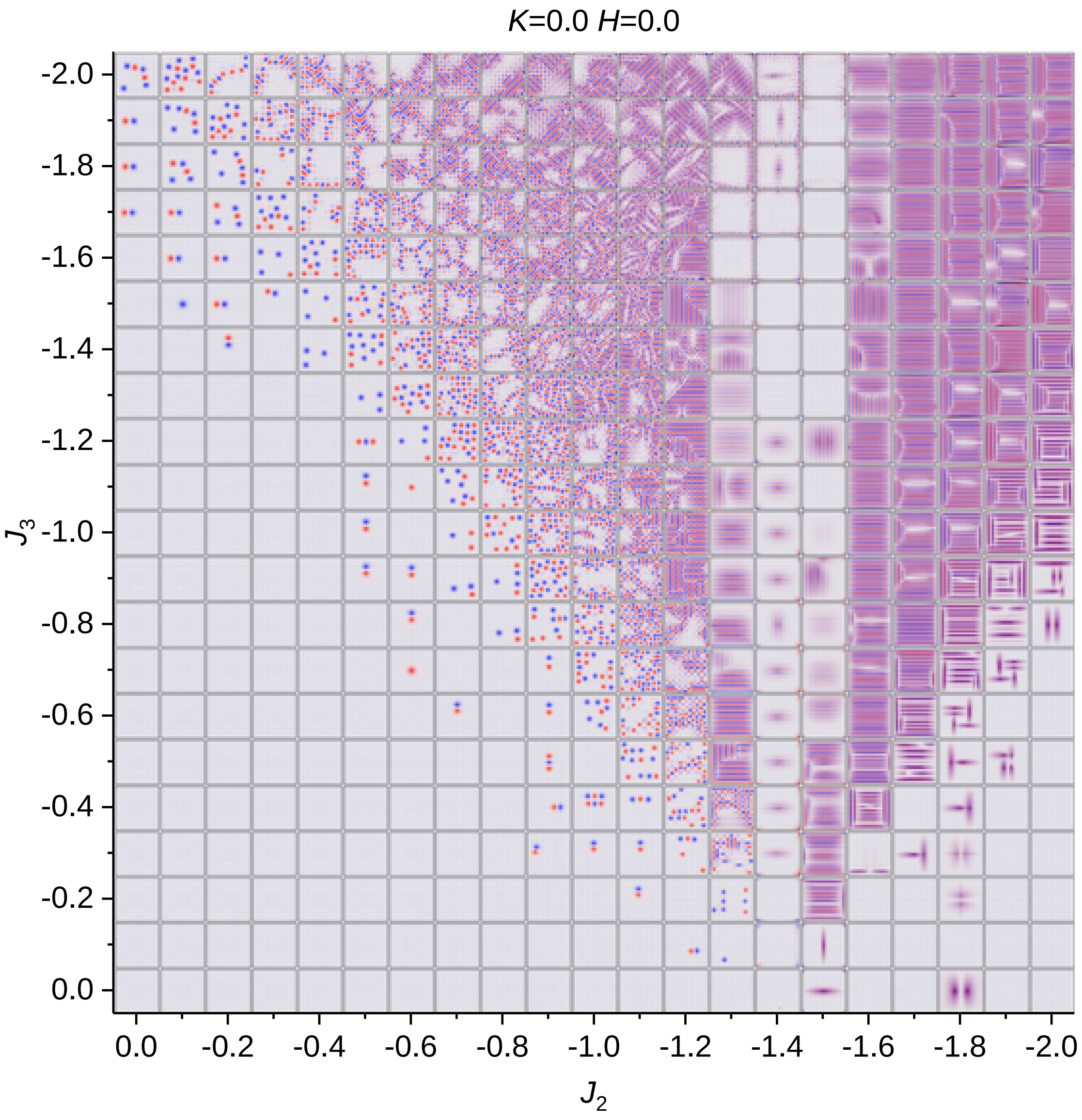

Supplementary Figure 3. **Typical metastable states (as functions of J_2 and J_3) obtained by relaxing the magnetic thin film with a random initial spin configuration in the presence of DDI.** The out-of-plane spin component is color-coded: blue is into the plane, red is out of the plane, white is in-plane. The model is a square element (40×40 spins) with the OBC. The fixed parameters (in units of $J_1 = 1$) are $K = 0$ and $H_z = 0$. The simulation is carried out by the OOMMF CG minimizer. By comparing Supplementary Figs. 3-6, it can be seen that at certain values of J_1, K and H_z, the values of J_2 and J_3 required for stabilizing frustrated skyrmions can be varied in a wide range. It is worth mentioning that in case of a smaller magnitude of J_3 is desired or reachable in materials, a larger magnitude of J_2 will be required for stabilizing the frustrated skyrmion, indicating the importance of the antiferromagnetic NNN and NNNN exchange interactions on the stabilization of the non-collinear spin texture in a ferromagnetic background.

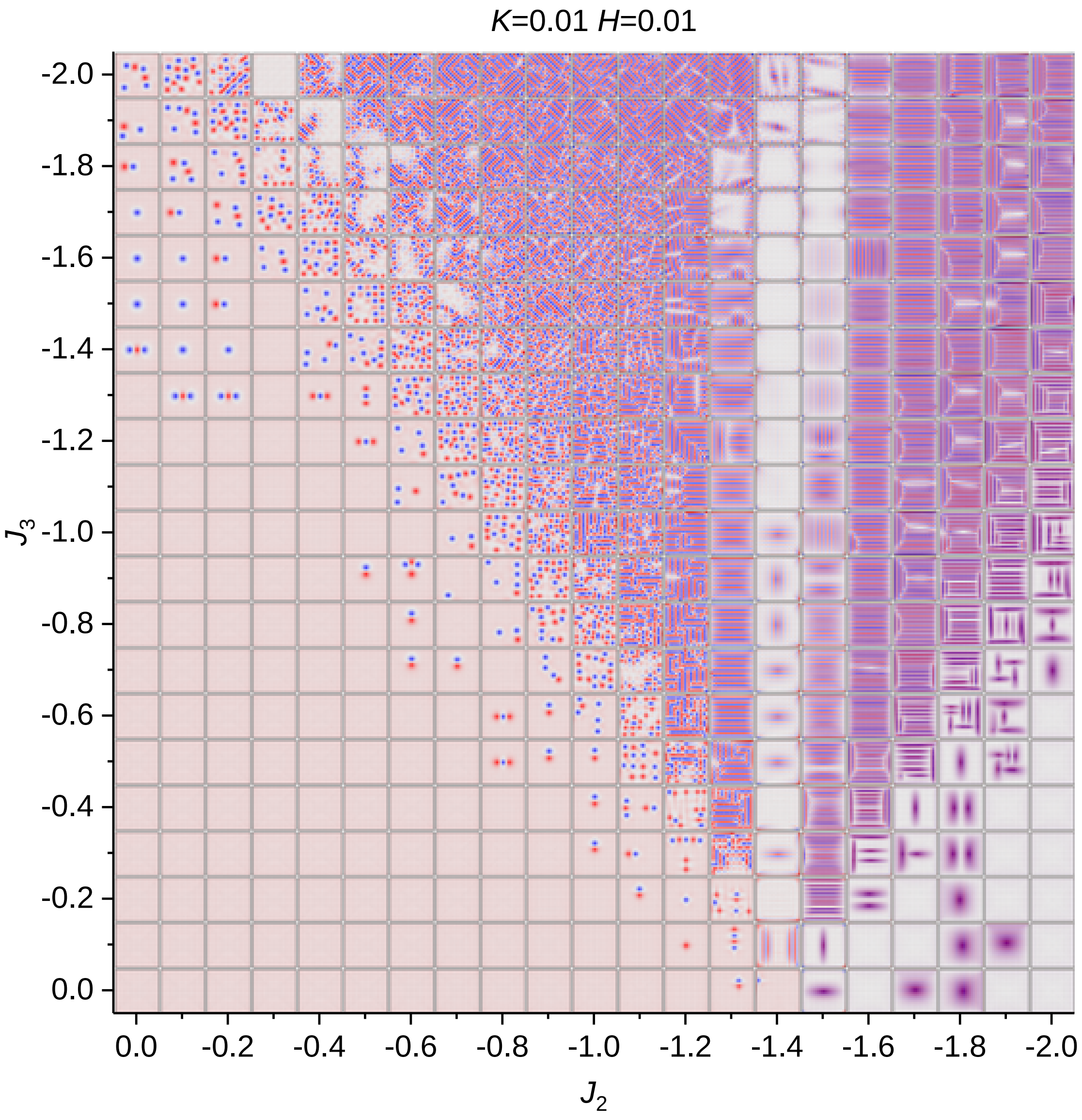

Supplementary Figure 4. **Typical metastable states (as functions of J_2 and J_3) obtained by relaxing the magnetic thin film with a random initial spin configuration in the presence of DDI.** The out-of-plane spin component is color-coded: blue is into the plane, red is out of the plane, white is in-plane. The model is a square element (40×40 spins) with the OBC. The fixed parameters (in units of $J_1 = 1$) are $K = 0.01$ and $H_z = 0.01$. The simulation is carried out by the OOMMF CG minimizer.

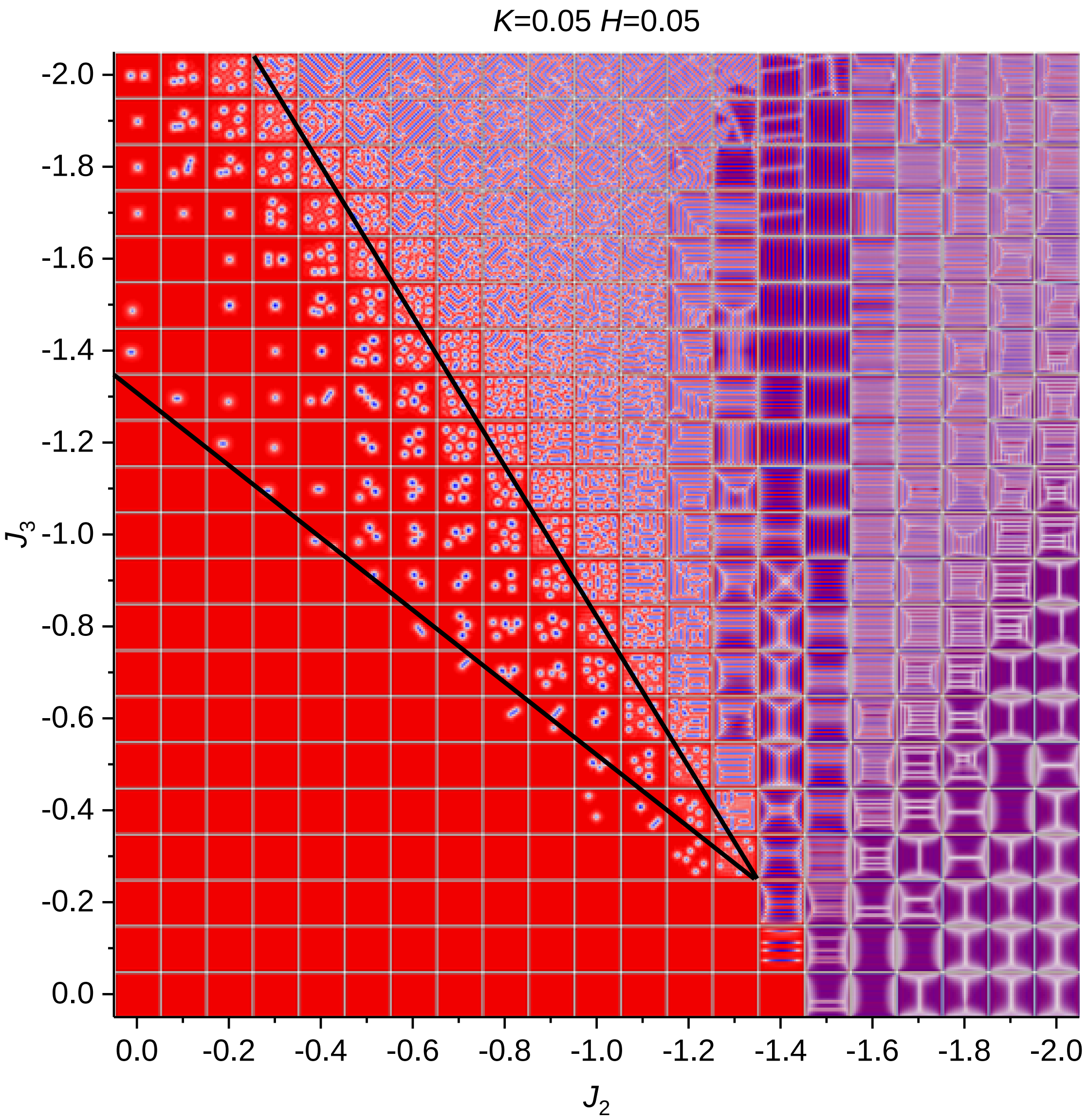

Supplementary Figure 5. **Typical metastable states (as functions of J_2 and J_3) obtained by relaxing the magnetic thin film with a random initial spin configuration in the presence of DDI.** The out-of-plane spin component is color-coded: blue is into the plane, red is out of the plane, white is in-plane. The model is a square element (40×40 spins) with the OBC. The fixed parameters (in units of $J_1 = 1$) are $K = 0.05$ and $H_z = 0.05$. The simulation is carried out by the OOMMF CG minimizer. Relaxed and isolated skyrmions and antiskyrmions appear in the region between the two black lines.

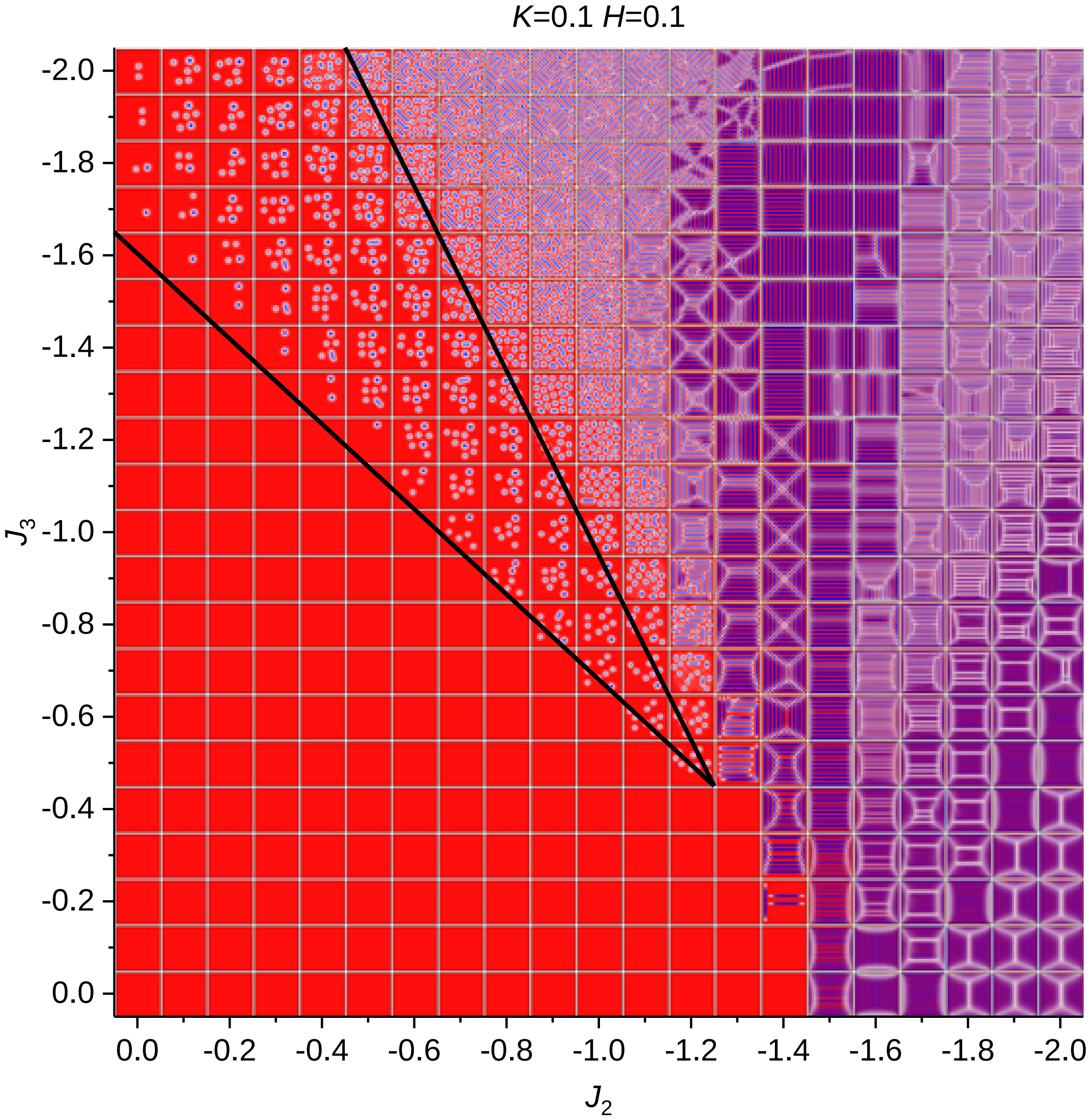

Supplementary Figure 6. **Typical metastable states (as functions of J_2 and J_3) obtained by relaxing the magnetic thin film with a random initial spin configuration in the presence of DDI.** The out-of-plane spin component is color-coded: blue is into the plane, red is out of the plane, white is in-plane. The model is a square element (40×40 spins) with the OBC. The fixed parameters (in units of $J_1 = 1$) are $K = 0.1$ and $H_z = 0.1$. The simulation is carried out by the OOMMF CG minimizer. Relaxed and isolated skyrmions and antiskyrmions appear in the region between the two black lines.

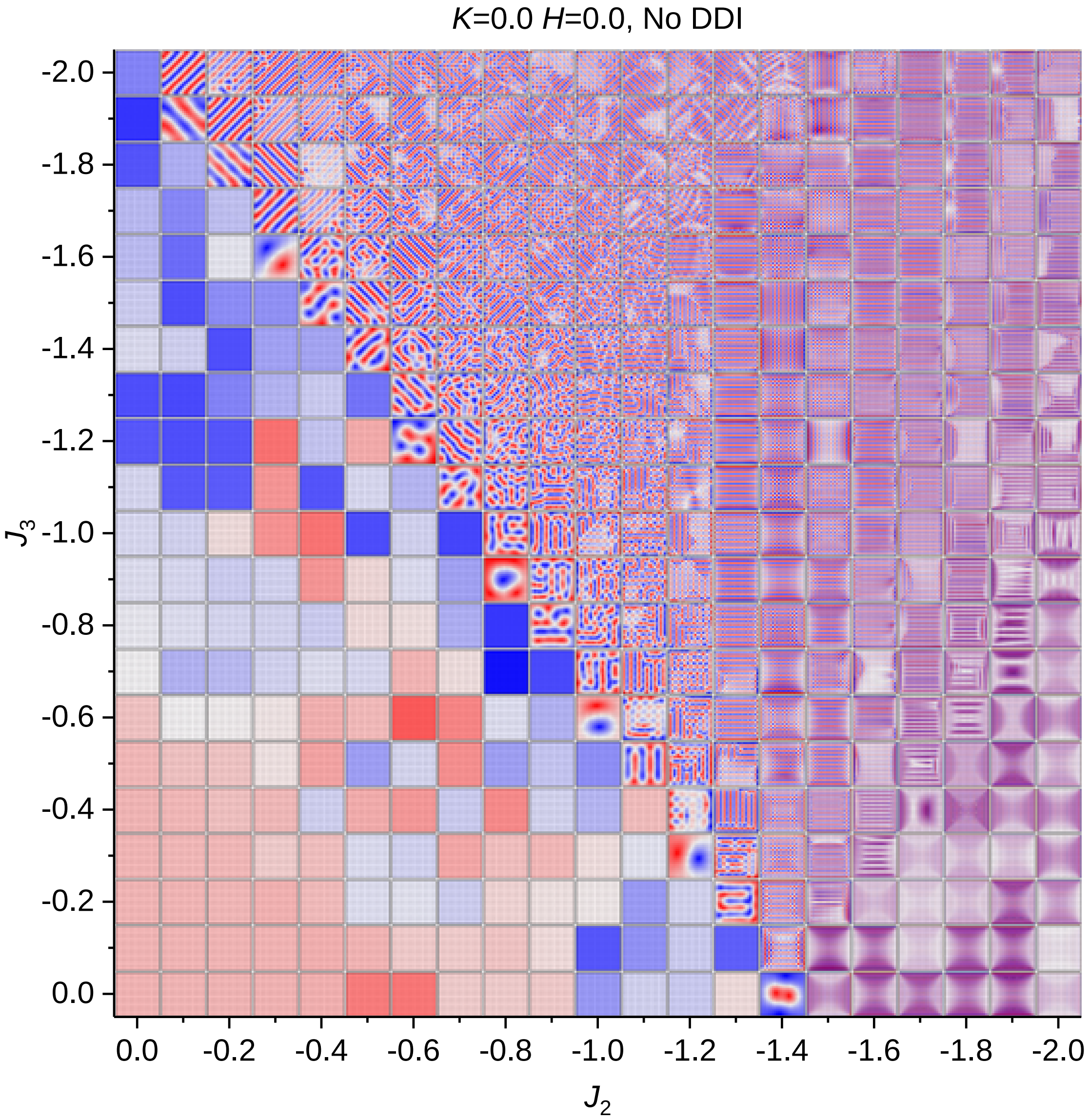

Supplementary Figure 7. **Typical metastable states (as functions of J_2 and J_3) obtained by relaxing the magnetic thin film with a random initial spin configuration in the absence of DDI.** The out-of-plane spin component is color-coded: blue is into the plane, red is out of the plane, white is in-plane. The model is a square element (40×40 spins) with the OBC. The fixed parameters (in units of $J_1 = 1$) are $K = 0$ and $H_z = 0$. The simulation is carried out by the OOMMF CG minimizer.

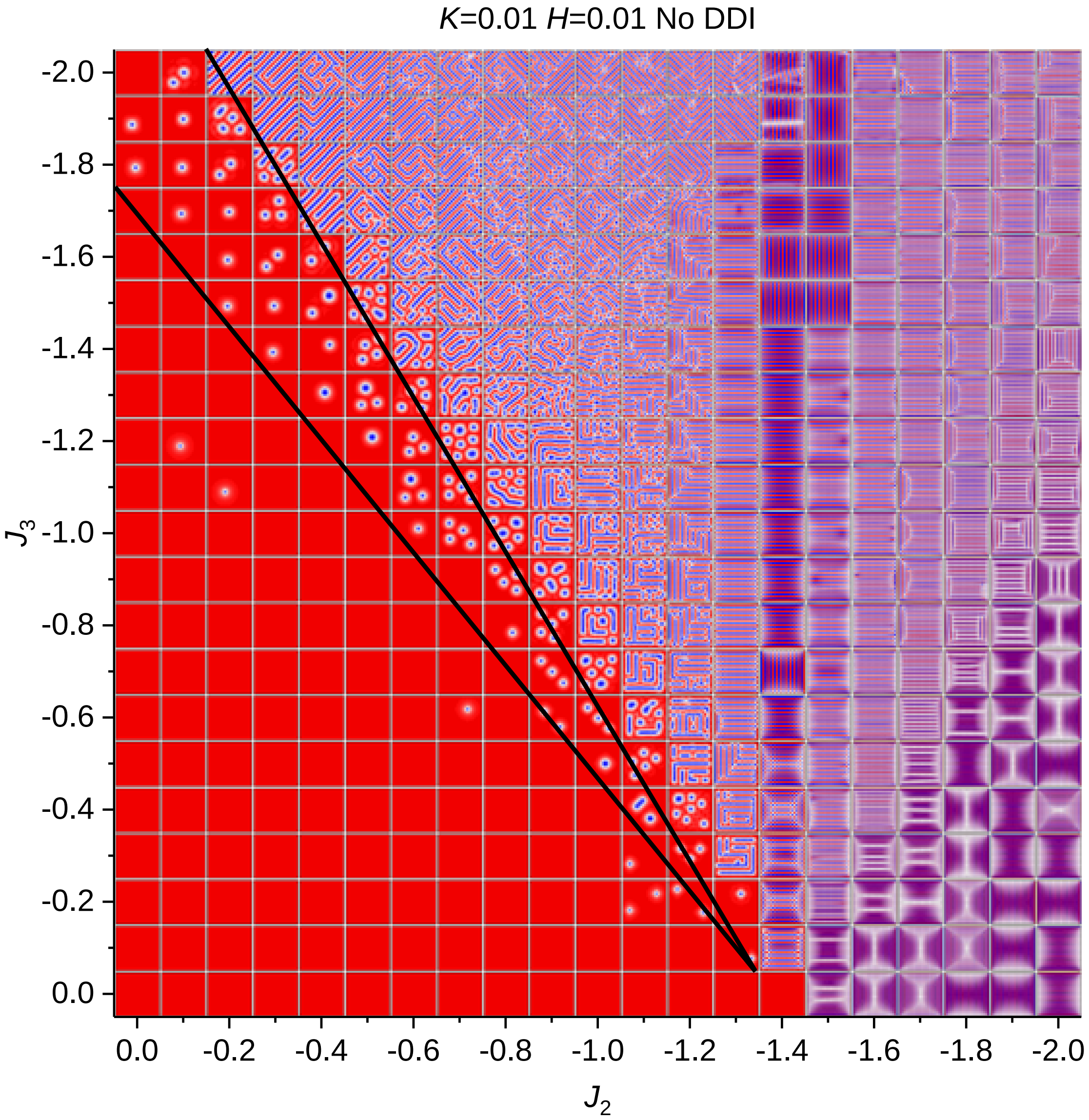

Supplementary Figure 8. Typical metastable states (as functions of J_2 and J_3) obtained by relaxing the magnetic thin film with a random initial spin configuration in the absence of DDI. The out-of-plane spin component is color-coded: blue is into the plane, red is out of the plane, white is in-plane. The model is a square element (40×40 spins) with the OBC. The fixed parameters (in units of $J_1 = 1$) are $K = 0.01$ and $H_z = 0.01$. The simulation is carried out by the OOMMF CG minimizer. Relaxed and isolated skyrmions and antiskyrmions appear in the region between the two black lines.

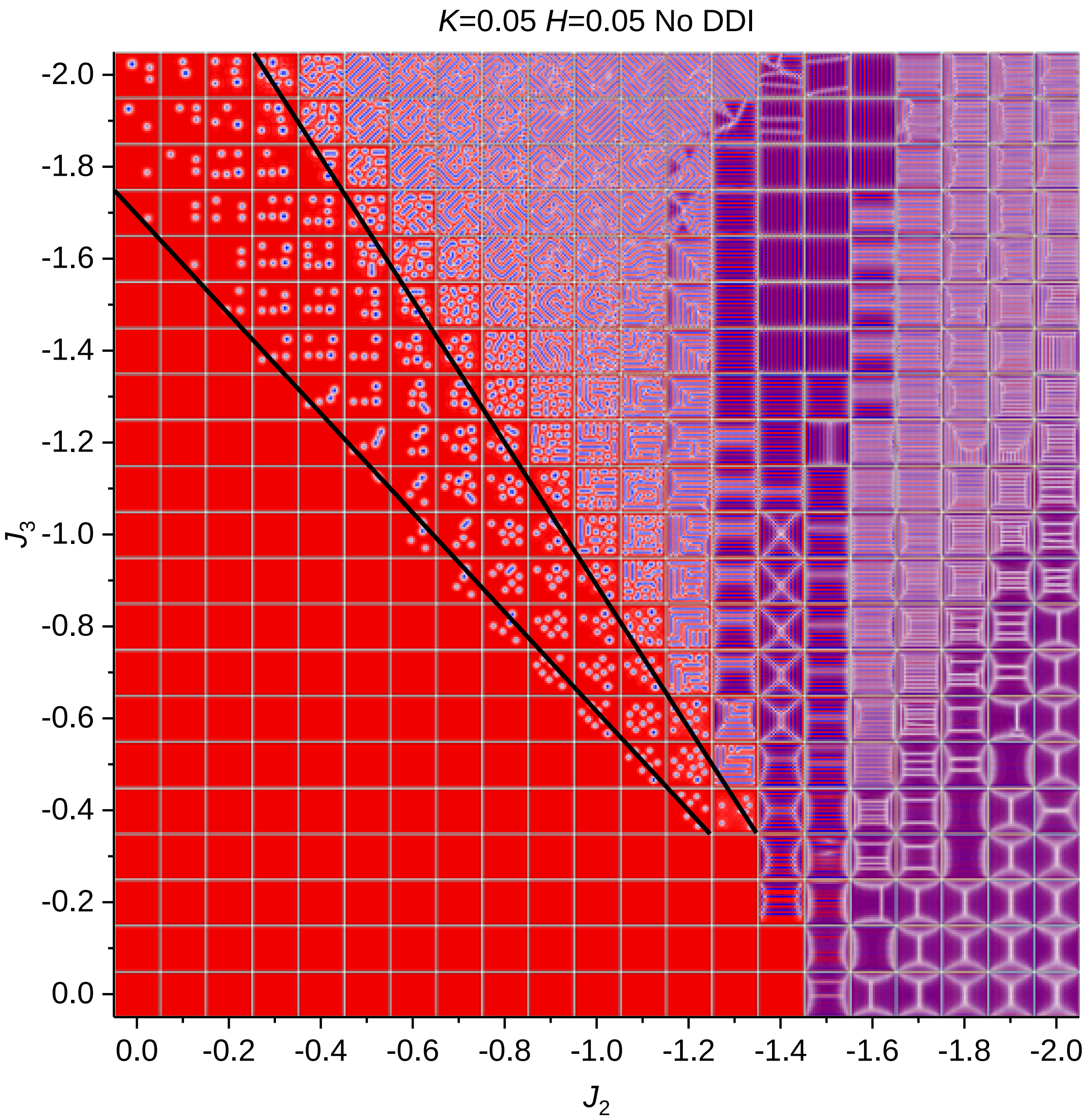

Supplementary Figure 9. **Typical metastable states (as functions of J_2 and J_3) obtained by relaxing the magnetic thin film with a random initial spin configuration in the absence of DDI.** The out-of-plane spin component is color-coded: blue is into the plane, red is out of the plane, white is in-plane. The model is a square element (40×40 spins) with the OBC. The fixed parameters (in units of $J_1 = 1$) are $K = 0.05$ and $H_z = 0.05$. The simulation is carried out by the OOMMF CG minimizer. Relaxed and isolated skyrmions and antiskyrmions appear in the region between the two black lines.

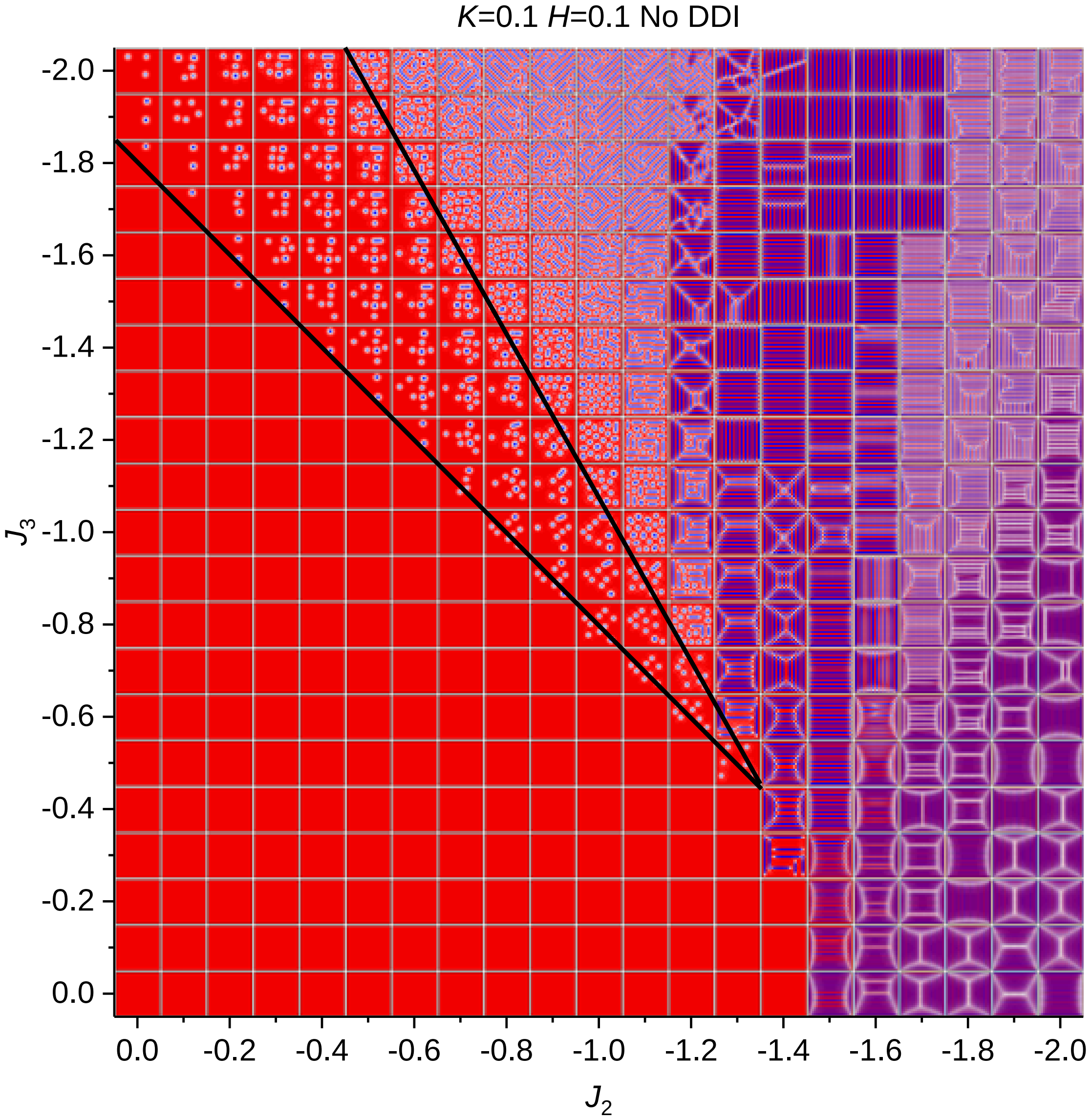

Supplementary Figure 10. **Typical metastable states (as functions of J_2 and J_3) obtained by relaxing the magnetic thin film with a random initial spin configuration in the absence of DDI.** The out-of-plane spin component is color-coded: blue is into the plane, red is out of the plane, white is in-plane. The model is a square element (40×40 spins) with the OBC. The fixed parameters (in units of $J_1 = 1$) are $K = 0.1$ and $H_z = 0.1$. The simulation is carried out by the OOMMF CG minimizer. Relaxed and isolated skyrmions and antiskyrmions appear in the region between the two black lines.

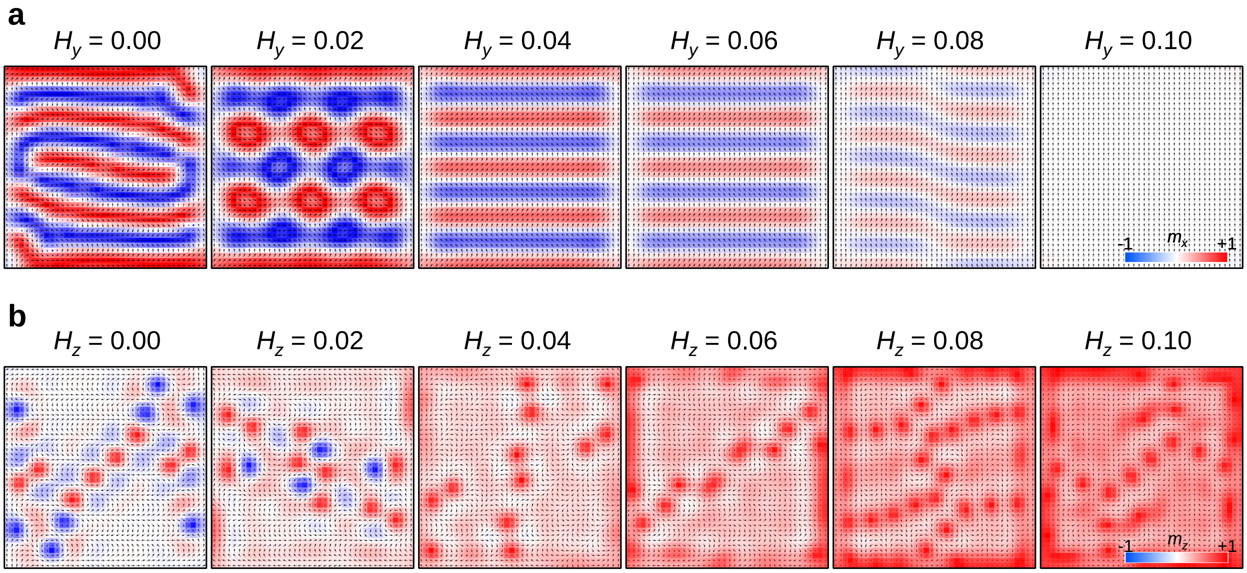

Supplementary Figure 11. **Relaxed spin configurations of a magnetic thin film** (40×40 **spins) with the OBC at (a) a certain in-plane applied field** H_y **and (b) a certain out-of-plane applied field** H_z**.** The out-of-plane spin component is color-coded: blue is into the plane, red is out of the plane, white is in-plane. The fixed parameters (in units of $J_1 = 1$) are $J_2 = -0.8$ and $J_3 = -1.2$, and $K = 0$. The simulation is carried out by the OOMMF CG minimizer. The edge spin configuration differs from the bulk spin configuration at certain applied fields due to the effects of OBC and DDI.

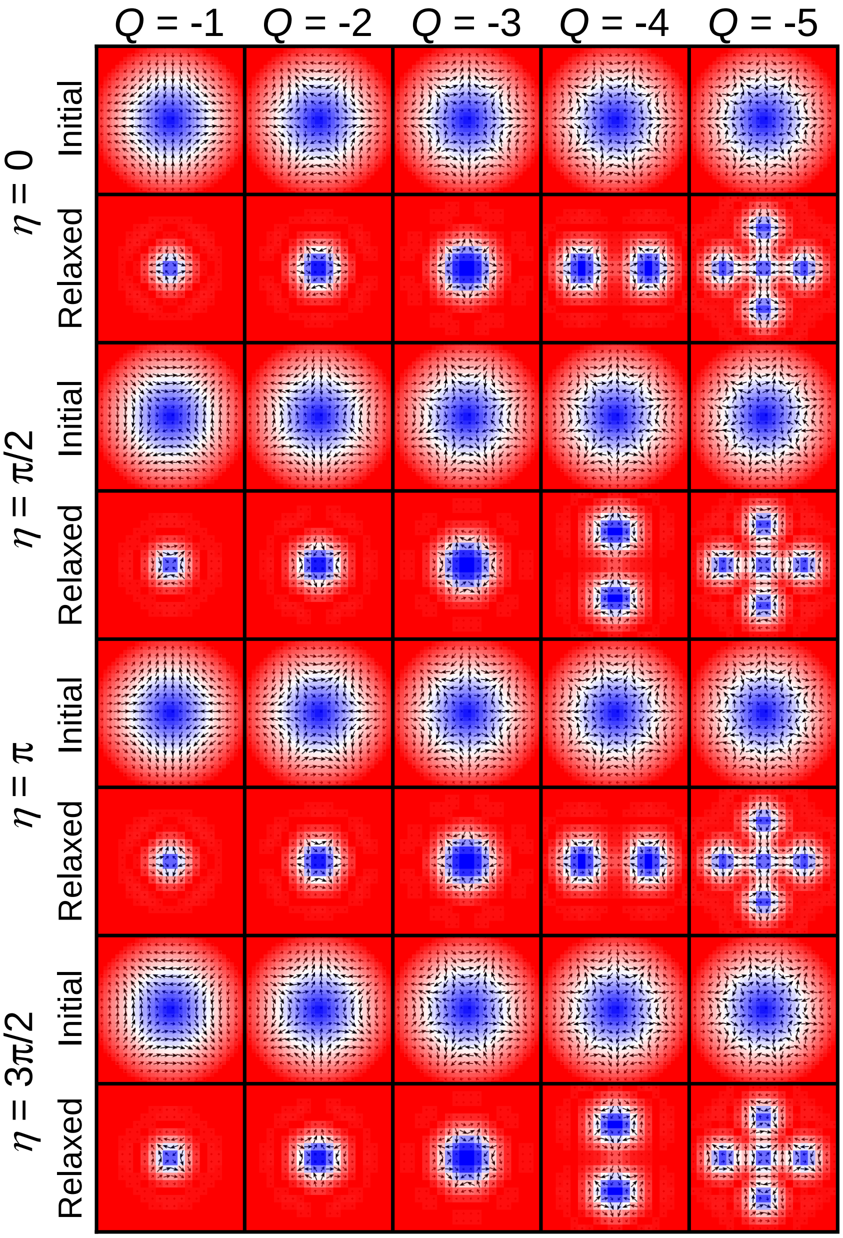

Supplementary Figure 12. **Relaxed states of antiskyrmions with different values of the skyrmion number Q and the helicity η.** We construct a radial symmetric antiskyrmion as the initial state. We check whether the initial geometry is preserved or broken after the relaxation. For the initial states of antiskyrmions with $Q = -1, -2, -3$, the numbers (Q, η) do not change after the relaxation although the radius shrinks. However, the antiskyrmion with $Q = -4$ is unstable and split into two antiskyrmions with $Q = -2$. The helicity η changes during this splitting process from that of the initial state with $\eta = \pm\pi/2$. The antiskyrmion with $Q = -5$ is also unstable and split into five antiskyrmions with $Q = -1$ and different η. The model is a square element with 40×40 spins. The parameters (in units of $J_1 = 1$) are $J_2 = -0.8$, $J_3 = -1.2$, $K = 0.1$, and $H_z = 0.1$. The simulation is carried out by the OOMMF CG minimizer.

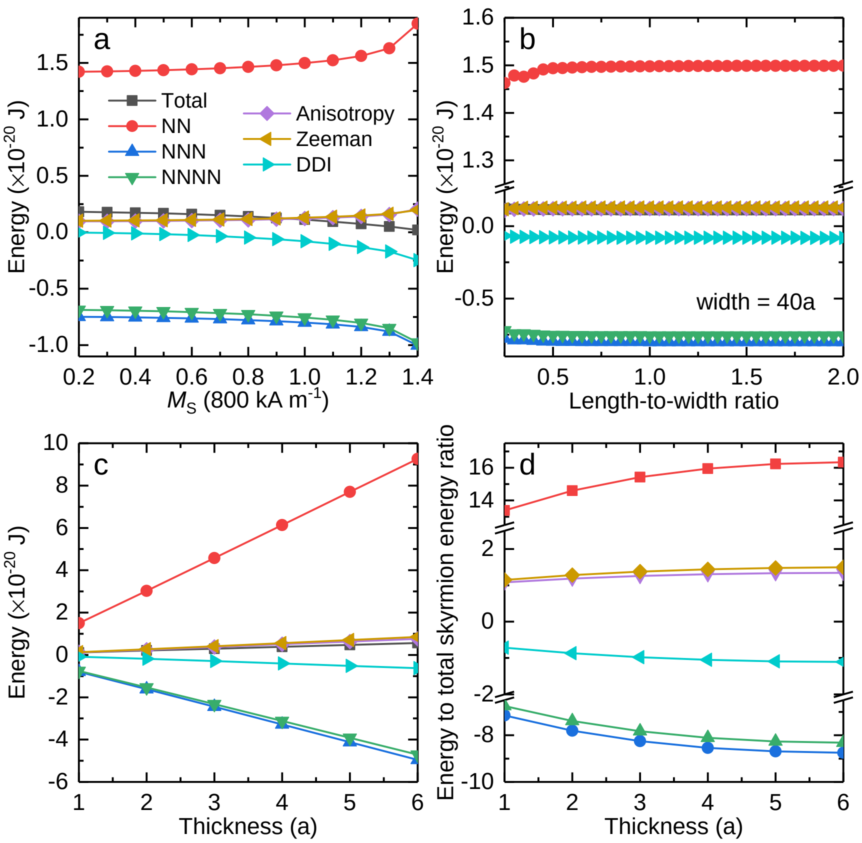

Supplementary Figure 13. **Skyrmion energy as a function of material and geometric parameters.** (**a**) Micromagnetic energy difference between the system with and without a skyrmion as a function of the saturation magnetization M_S of the magnetic thin film, of which the length, width, and thickness are fixed at $40a$, $40a$, and a, respectively. (**b**) Micromagnetic energy difference between the system with and without a skyrmion as a function of the length-to-width ratio of the magnetic thin film, of which the width and thickness are fixed at $40a$ and a, respectively. (**c**) Micromagnetic energy difference between the system with and without a skyrmion as a function of the thickness of the magnetic thin film, of which the length and width are fixed at $40a$. (**d**) Energy to total skyrmion energy ratio as a function of the thickness of the magnetic thin film, of which the length and width are fixed at $40a$. In all simulations, the fixed parameters (in units of $J_1 = 1$) are $J_2 = -0.8$, $J_3 = -1.2$, $K = 0.1$, and $H_z = 0.1$. For the system with the skyrmion, a ground-state skyrmion with $Q = 1$ and $\eta = \pi/2$ is placed at the film center and the given system is relaxed. For the system without the skyrmion, the relaxed state of the film is a ferromagnetic state with spins pointing along the $+z$ direction. The energy difference between the systems with and without a skyrmion can be seen as the energy of the skyrmion. The black, red, blue, green, purple, yellow, and turquoise curves denote the differences of the total energy, NN exchange energy, NNN exchange energy, NNNN exchange energy, anisotropy energy, Zeeman energy, and DDI energy, respectively. It shows that the skyrmion energy does not change with the length-to-width ratio. On the other hand, the NN exchange energy of a skyrmion increases with increasing thickness, while the NNN exchange, NNNN exchange, and DDI energies of a skyrmion decrease with increasing thickness. The Zeeman, anisotropy, and total energies of a skyrmion only slightly increases with increasing thickness. Besides, it can be seen the skyrmion energy varies with increasing M_S, especially when M_S is larger than a certain value.

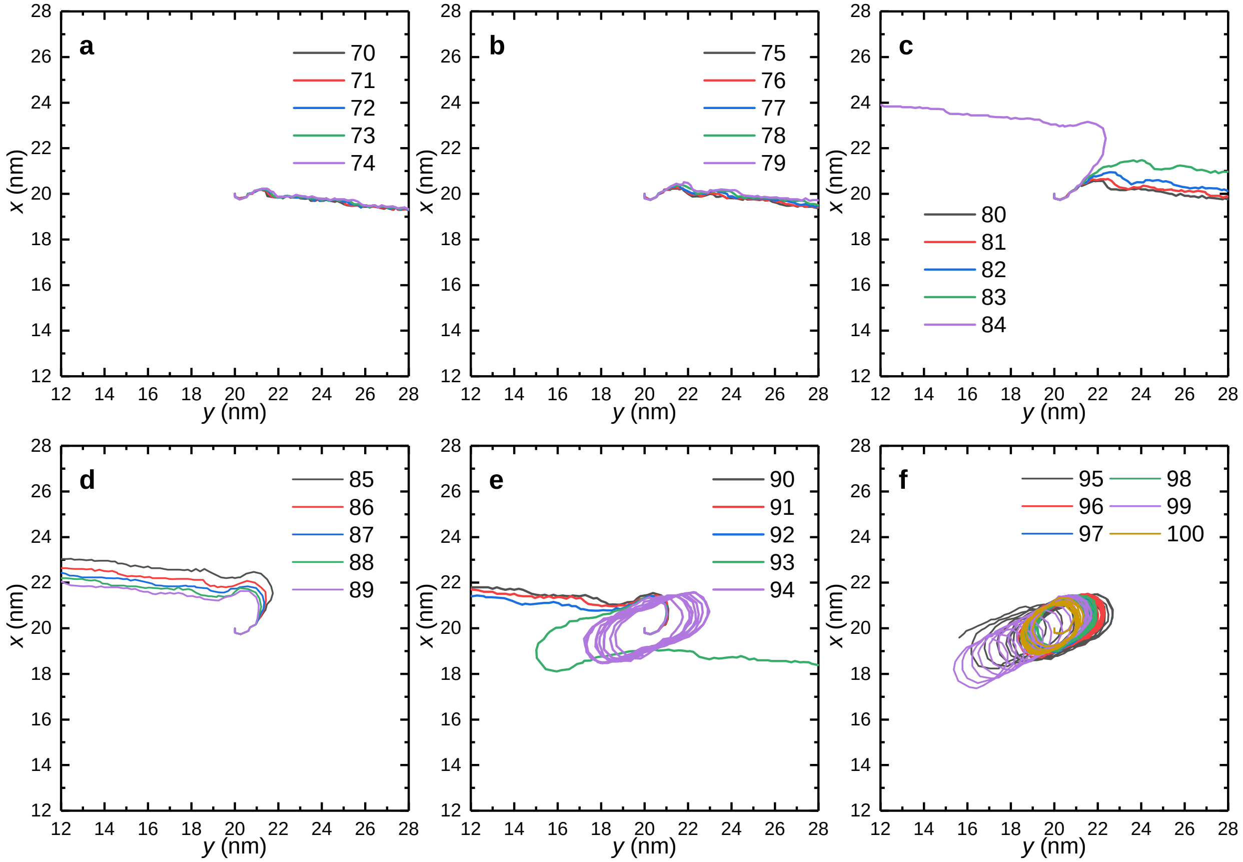

Supplementary Figure 14. **Trajectories of the skyrmion at varied values of the driving current density.** The skyrmion has an initial helicity number of $\eta = \pi/2$. The driving current density ranges from $j = 70 \times 10^{10}$ A m^{-2} to $j = 100 \times 10^{10}$ A m^{-2}. (**a**) At small driving current densities (e.g., $j = 70 \times 10^{10}$ A m^{-2}), the skyrmion moves toward the right. (**c**) When the driving current density is increased to a larger value (e.g., $j = 84 \times 10^{10}$ A m^{-2}), the helicity unlocking event occurs once, and (**d**) the skyrmion moves toward the left after the flip of the helicity. (**f**) When the driving current density is further increased to a more larger value (e.g., $j = 94 \times 10^{10}$ A m^{-2}), the helicity is totally unlocked, and the skyrmion moves in an orbital circle. The model is a square element with 100×100 spins. The parameters (in units of $J_1 = 1$) are $J_2 = -0.8$, $J_3 = -1.2$, $K = 0.1$, $H_z = 0.1$, and $\alpha = 0.1$. The simulation is carried out by the OOMMF solver integrating the LLG equation including the spin torque. The unit of the current density is 10^{10} A m^{-2}.

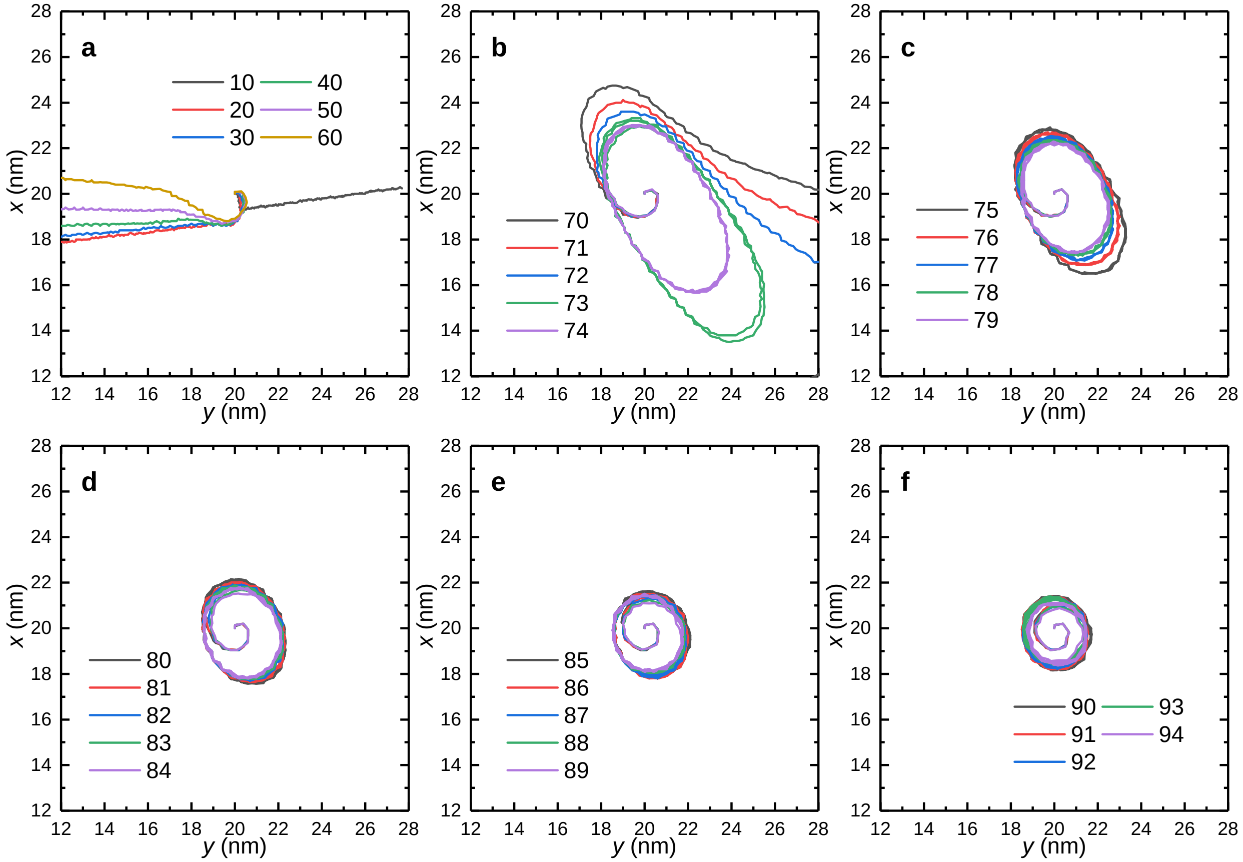

Supplementary Figure 15. **Trajectories of the antiskyrmion at varied values of the driving current density.** The antiskyrmion has an initial helicity number of $\eta = \pi/2$. The driving current density ranges from $j = 10 \times 10^{10}$ A m^{-2} to $j = 94 \times 10^{10}$ A m^{-2}. (**a**) At small driving current densities (e.g., $j = 10 \times 10^{10}$ A m^{-2}), the antiskyrmion moves toward the right. (**b**) When the driving current density is increased to a larger value (e.g., $j = 20 \times 10^{10}$ A m^{-2}), the helicity unlocking event occurs once, and the antiskyrmion moves toward the left after the flip of the helicity. (**c**)-(**f**) When the driving current density is further increased to a more larger value (e.g., $j = 73 \times 10^{10}$ A m^{-2}), the helicity is totally unlocked, and the antiskyrmion moves in an orbital circle. The model is a square element with 100×100 spins. The parameters (in units of $J_1 = 1$) are $J_2 = -0.8$, $J_3 = -1.2$, $K = 0.1$, $H_z = 0.1$, and $\alpha = 0.1$. The simulation is carried out by the OOMMF solver integrating the LLG equation including the spin torque. The unit of the current density is 10^{10} A m^{-2}.

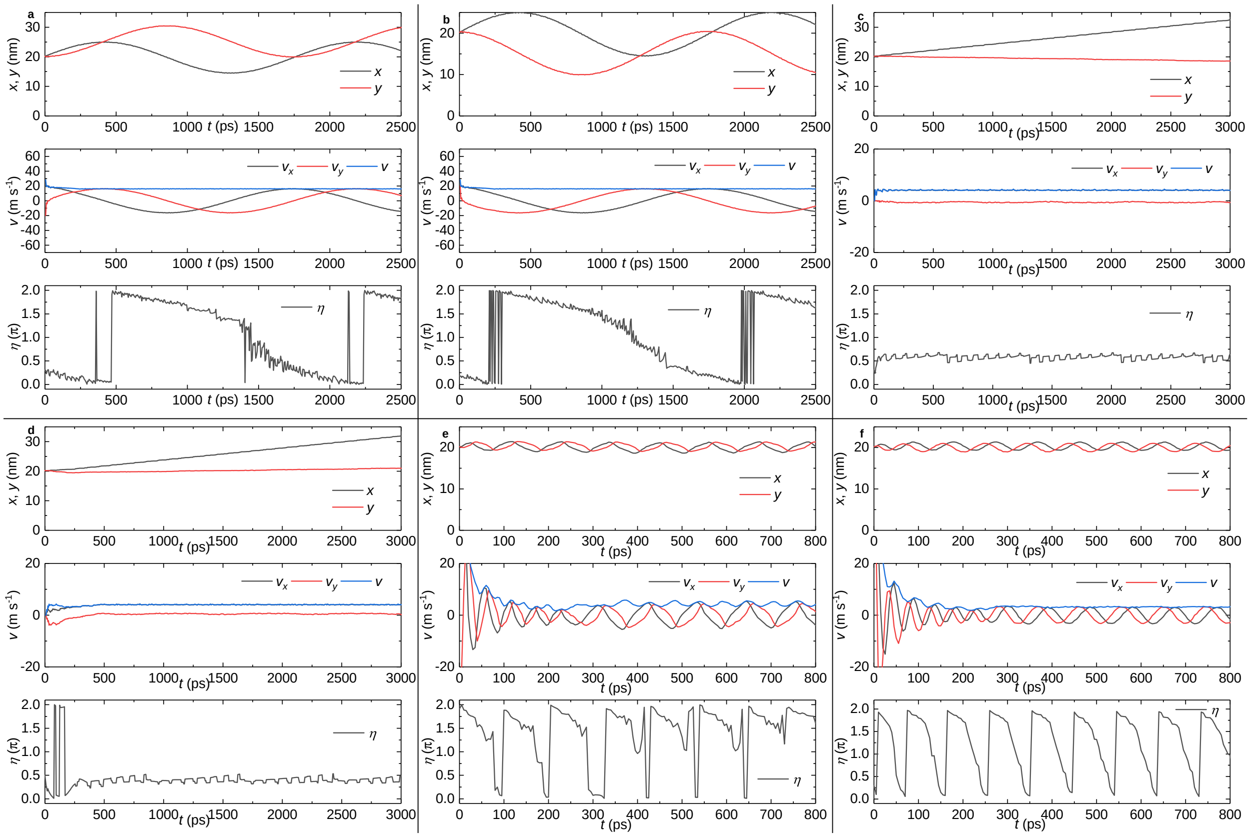

Supplementary Figure 16. **The time dependences of the location** (x, y)**, the velocity** (v_x, v_y, $v = \sqrt{v_x^2 + v_y^2}$)**, and the helicity** (η) **corresponding to Fig. 6 in the main text.** (**a**) and (**b**) are corresponding to Fig. 6(a) and 6(b), respectively, where $j = 50 \times 10^{10}$ A m^{-2}. (**c**) and (**d**) are corresponding to Fig. 6(c) and 6(d), respectively, where $j = 10 \times 10^{10}$ A m^{-2}. (**e**) and (**f**) are corresponding to Fig. 6(e) and 6(f), respectively, where $j = 100 \times 10^{10}$ A m^{-2}. The helicity in (**a**) and (**b**) is unlocked due to the absence of DDI. The helicity in (**c**) and (**d**) is locked due to the presence of DDI. The helicity in (**e**) and (**f**) is unlocked due to the presence of both DDI and a strong driving current. The helicity number is calculated by $\eta = \arctan(m_y/m_x)$ on the extreme right spin at the $m_z = 0$ circle of a skyrmion or an antiskyrmion.

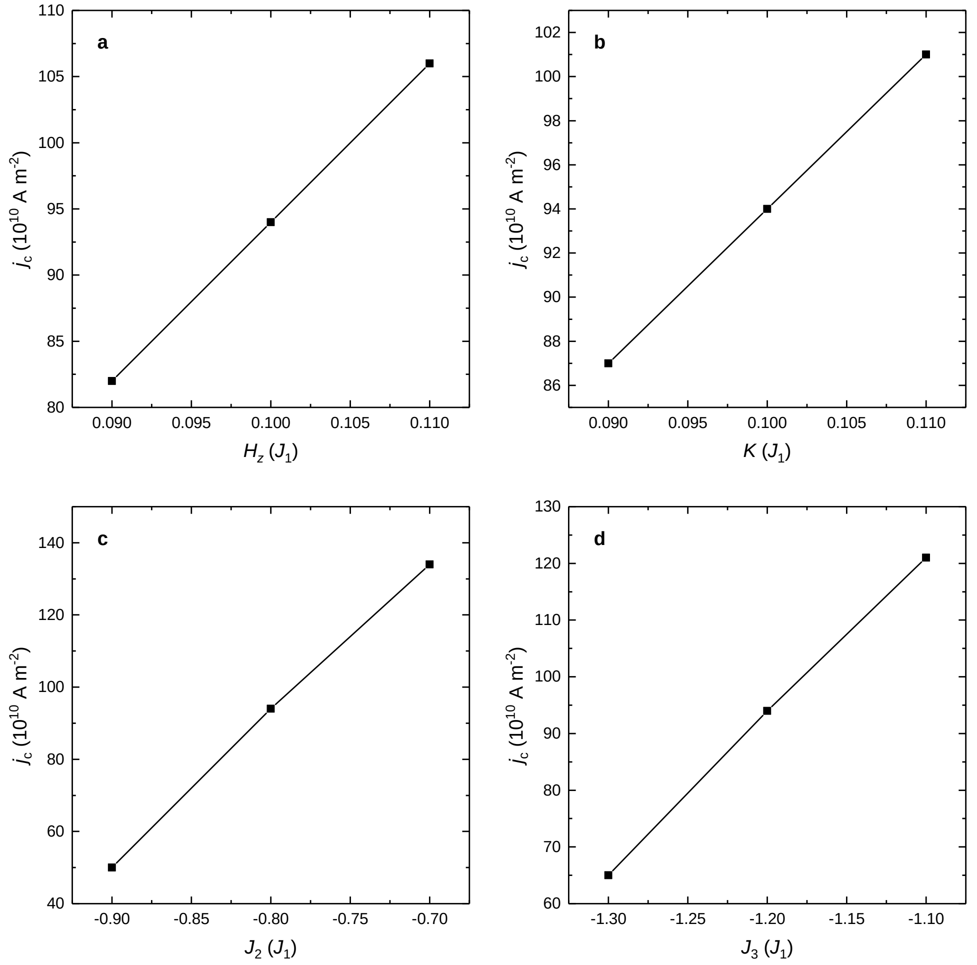

Supplementary Figure 17. **Critical current density j_c for the helicity locking-unlocking transition as a function of (a) H_z, (b) K, (c) J_2, and (d) J_3.** The model is a square element with 100×100 spins. The default parameters (in units of $J_1 = 1$) are $J_2 = -0.8$, $J_3 = -1.2$, $K = 0.1$, $H_z = 0.1$, and $\alpha = 0.1$. The simulation is carried out by the OOMMF solver integrating the LLG equation including the spin torque.

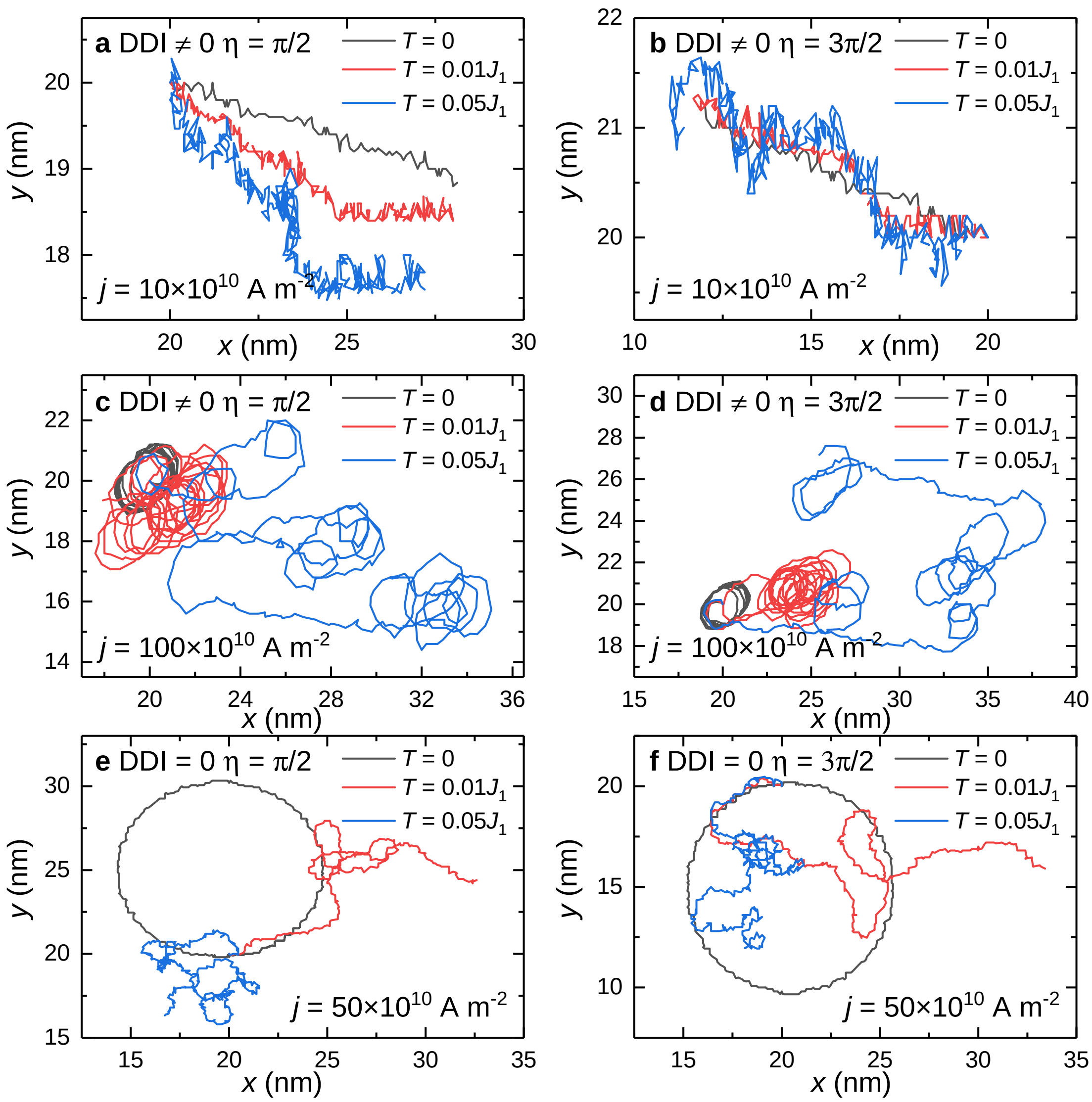

Supplementary Figure 18. **Thermal effect on the motion dynamics of the frustrated skyrmion.** (**a**, **b**) Temperature-dependent trajectories of skyrmions with $\eta = \pi/2, 3\pi/2$ driven by a small current ($j = 10 \times 10^{10}$ A m^{-2}) in the presence of DDI. (**c**, **d**) Temperature-dependent trajectories of skyrmions with $\eta = \pi/2, 3\pi/2$ driven by a large current ($j = 100 \times 10^{10}$ A m^{-2}) in the presence of DDI. (**e**, **f**) Temperature-dependent trajectories of skyrmions with $\eta = \pi/2, 3\pi/2$ driven by a moderate current ($j = 50 \times 10^{10}$ A m^{-2}) in the absence of DDI. The model is a square element with 100×100 spins. The parameters (in units of $J_1 = 1$) are $J_2 = -0.8$, $J_3 = -1.2$, $K = 0.1$, $H_z = 0.1$, $\alpha = 0.1$. The simulation is carried out by the OOMMF solver integrating the LLG equation including the spin torque. In the presence of DDI and thermal effect, the skyrmion driven by a small current with locked helicity moves toward a certain direction (depending on η), however, the trajectory is fluctuated and the skyrmion shows Brownian motion behavior. Besides, the skyrmion driven by a large current with unlocked helicity shows rotational motion associated with Brownian motion behavior. When the DDI is removed, the trajectory of the skyrmion at finite temperature is rather fluctuated, which also shows a mixed dynamics of rotational motion and Brownian motion.

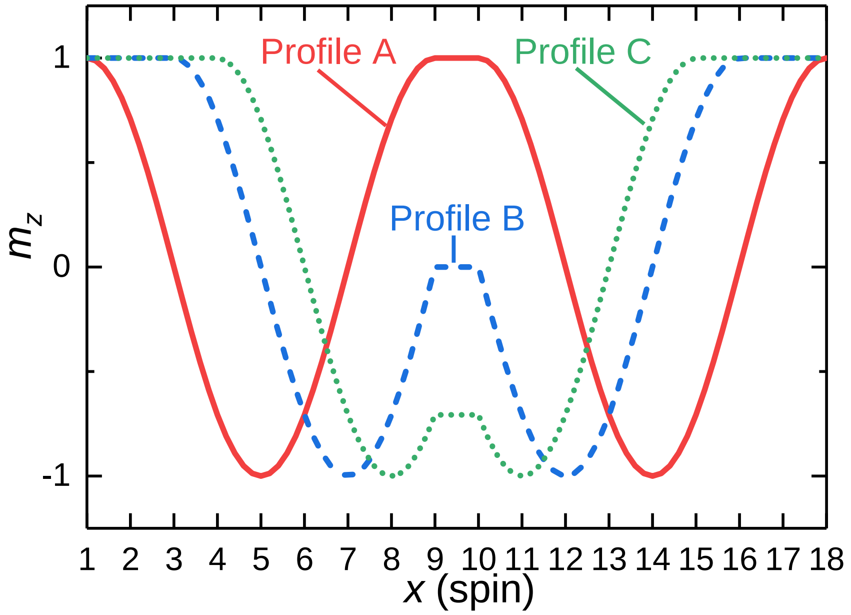

Supplementary Figure 19. Initial spin configuration (m_z component) for the study of the skyrmion-skyrmion, antiskyrmion-antiskyrmion, and skyrmion-antiskyrmion interactions. The model is a magnetic rectangular element with 18×9 spins with the OBC. We construct two skyrmions (antiskyrmions) with $Q = \pm 1$ and different η placed at the left and right sides of the sample, respectively, with three types of initial spin configuration. The initial spin configuration of profile A assumes that the two skyrmions have no overlap at the initial state, which is used to study the natural interaction between the two skyrmions. The initial spin configuration of profile B assumes that the two skyrmions have a moderate overlap at the initial state, which is used to study the interaction between the two skyrmions when they are colliding each other driving by moderate external driving forces. The initial spin configuration of profile C assumes that the two skyrmions have a strong overlap at the initial state, which is used to study the interaction between the two skyrmions when they are colliding each other driving by strong external driving forces.

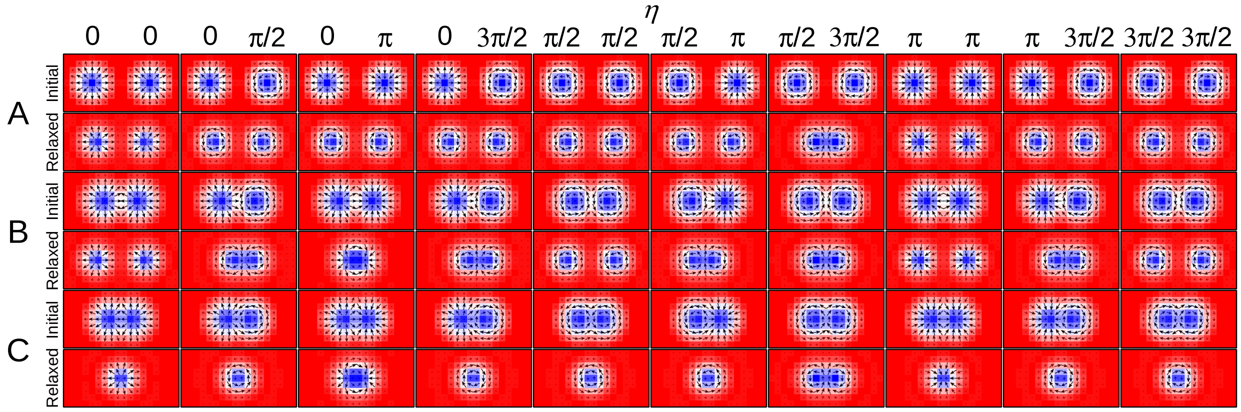

Supplementary Figure 20. **Skyrmion-skyrmion interaction.** Two skyrmions and different η are placed at the left and right sides of the magnetic rectangular element with three types of initial spin configuration (cf. Supplementary Fig. 19). When the initial spin configuration of profile A is employed, the skyrmion with $\eta = \pi/2$ and the skyrmion with $\eta = 3\pi/2$ merge into one skyrmion with $Q = 2$, indicating the natural attractions between these two skyrmions. When the initial spin configuration of profile B is employed, the two skyrmions with unequal η merge into a skyrmion with $Q = 2$, while the two skyrmions with identical η cannot merge into one skyrmion with $|Q| > 1$, indicating the natural repulsions between the two skyrmions with identical η. When the initial spin configuration of profile C is employed, the skyrmion with $\eta = 0$ and the skyrmion with $\eta = \pi$ merge into one skyrmion with $Q = 2$. The skyrmion with $\eta = \pi/2$ and the skyrmion with $\eta = 3\pi/2$ merge into one skyrmion with $Q = 2$. However, for other cases, the relaxed state is only one skyrmion with $Q = 1$. This indicates that a strong collision between two skyrmions may lead to the annihilation of one skyrmion, unless one has $\eta = 0$ and the other has $\eta = \pi$ or one has $\eta = \pi/2$ and the other has $\eta = 3\pi/2$. The model is a magnetic rectangular element with 18×9 spins. The parameters (in units of $J_1 = 1$) are $J_2 = -0.8$, $J_3 = -1.2$, $K = 0.1$, and $H_z = 0.1$. The simulation is carried out by the OOMMF CG minimizer.

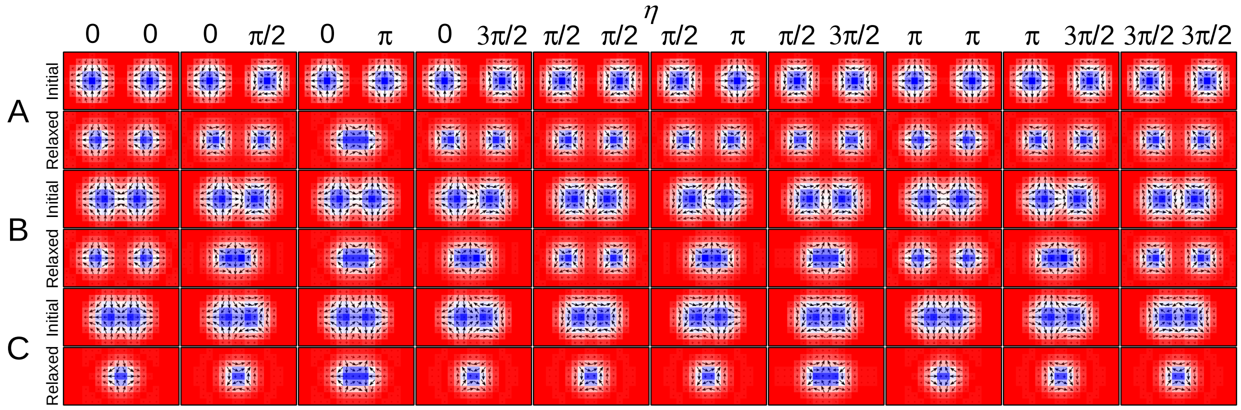

Supplementary Figure 21. **Antiskyrmion-antiskyrmion interaction.** The geometric and material parameters are the same as those used in Supplementary Fig. 20. Two antiskyrmions and different η are placed at the left and right sides of the magnetic rectangular element with three types of initial spin configuration (cf. Supplementary Fig. 19). When the initial spin configuration of profile A is employed, the antiskyrmion with $\eta = 0$ and the antiskyrmion with $\eta = \pi$ merge into one antiskyrmion with $Q = -2$, indicating the natural attractions between these two antiskyrmions. When the initial spin configuration of profile B is employed, the two antiskyrmions with unequal η merge into an antiskyrmion with $Q = -2$, while the two antiskyrmions with identical η cannot merge into one antiskyrmion with $|Q| > 1$, indicating the natural repulsions between the two antiskyrmions with identical η. When the initial spin configuration of profile C is employed, the antiskyrmion with $\eta = 0$ and the antiskyrmion with $\eta = \pi$ merge into one antiskyrmion with $Q = -2$. The antiskyrmion with $\eta = \pi/2$ and the antiskyrmion with $\eta = 3\pi/2$ merge into one antiskyrmion with $Q = -2$. However, for other cases, the relaxed state is only one antiskyrmion with $Q = -1$. This indicates that a strong collision between two antiskyrmions may lead to the annihilation of one antiskyrmion, unless one has $\eta = 0$ and the other has $\eta = \pi$, or one has $\eta = \pi/2$ and the other has $\eta = 3\pi/2$.

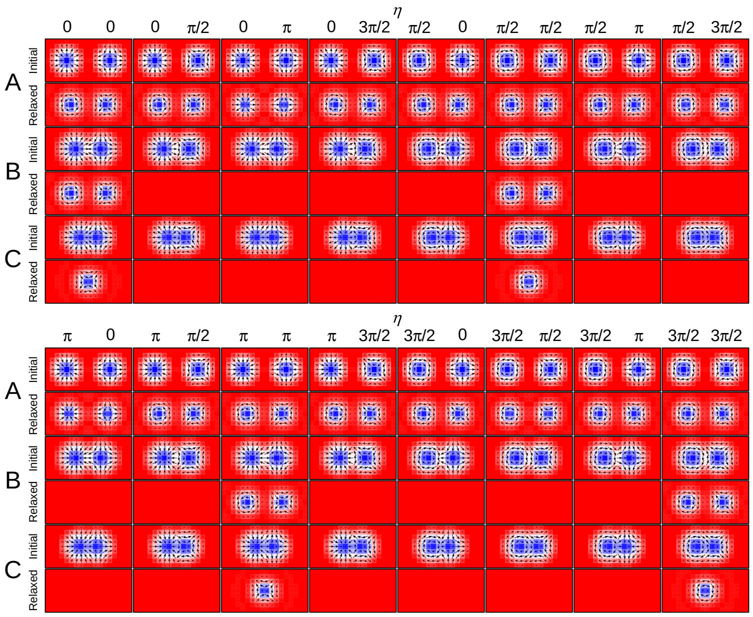

Supplementary Figure 22. **Skyrmion-antiskyrmion interaction.** The geometric and material parameters are the same as those used in Supplementary Fig. 20. A skyrmion ($Q = 1$) and an antiskyrmion ($Q = -1$) with different η are placed, respectively, at the left and right sides of the magnetic rectangular element with three types of initial spin configuration (cf. Supplementary Fig. 19). When the initial spin configuration of profile A is employed, there is no attraction between the skyrmion and the antiskyrmion. The η of the relaxed skyrmion and the antiskyrmion will be altered to the case with lower energy, unless the skyrmion and the antiskyrmion: i. have identical $\eta = \pi/2$ or $\eta = 3\pi/2$; ii. one has $\eta = 0$ and the other has $\eta = \pi$; iii. one has $\eta = \pi/2$ and the other has $\eta = 3\pi/2$. When the initial spin configuration of profile B is employed, the skyrmion and the antiskyrmion will process a pair annihilation unless they have identical η. The skyrmion and the antiskyrmion have identical η will repel each other. Similarly, when the initial spin configuration of profile C is employed, the skyrmion and the antiskyrmion will process a pair annihilation unless they have identical η. If the skyrmion and the antiskyrmion have identical $\eta = 0$ or $\eta = \pi$, the skyrmion will be annihilated, while if the skyrmion and the antiskyrmion have identical $\eta = \pi/2$ or $\eta = 3\pi/2$, the antiskyrmion will be annihilated. The results indicate that a moderate or strong collision between a skyrmion and an antiskyrmion will lead to the pair annihilation of the skyrmion and the antiskyrmion, unless they have identical η where the annihilation of only the skyrmion or only the antiskyrmion will be processed under a strong collision.

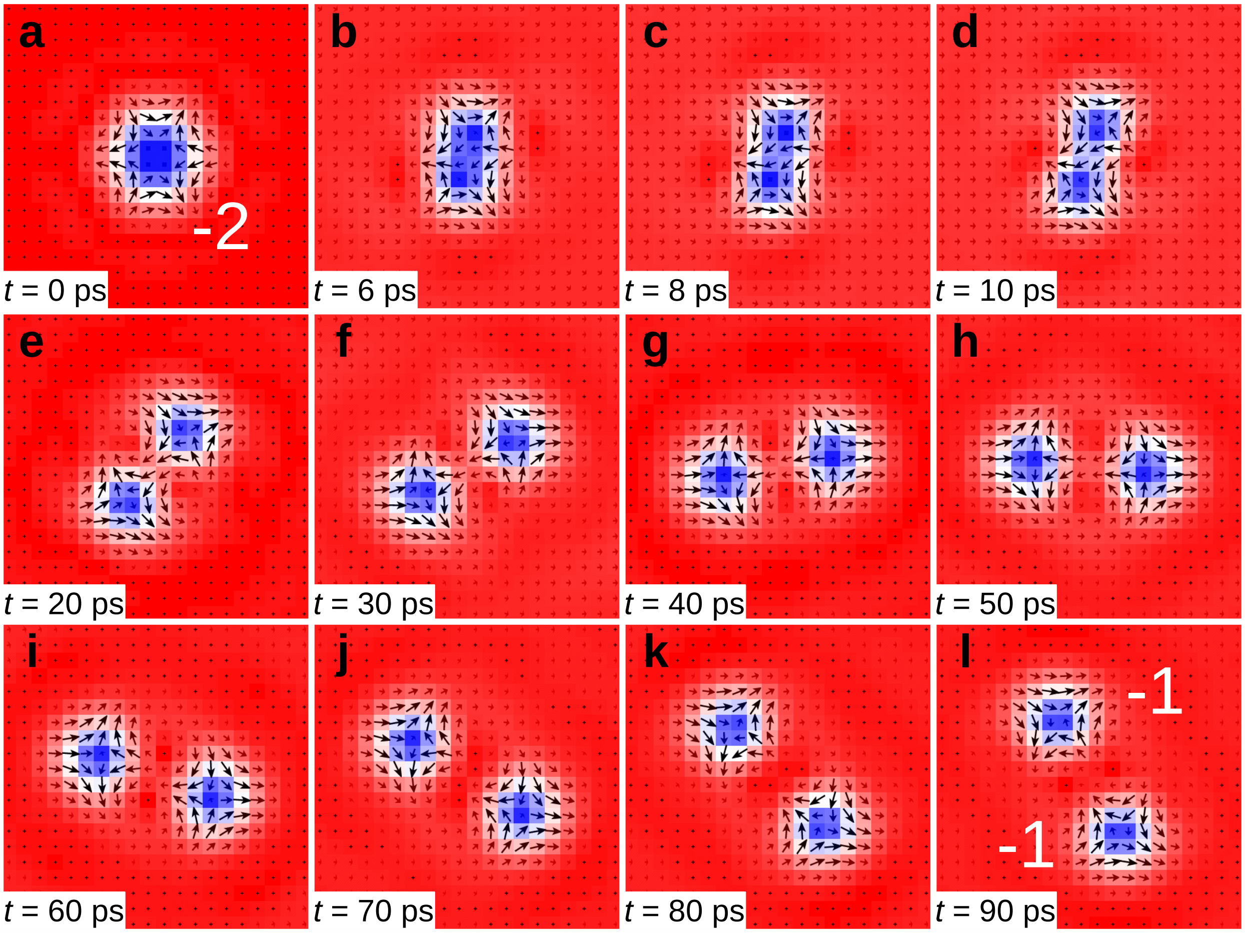

Supplementary Figure 23. **Forced separation of two antiskyrmions from a bi-antiskyrmion.** The skyrmion number Q is indicated in (**a**) and (**l**). We first place a relaxed bi-antiskyrmion at the center of the sample. Then, a driving current is vertically injected to the sample, which can be realized by the spin Hall effect in a heavy-metal substrate. It is found that the bi-antiskyrmion starts to rotate counterclockwise, and forms a clear peanut-like shape, as shown in (**d**). When it has rotated almost 90 degrees, two antiskyrmions are almost generated, as shown in (**h**). As shown in (**l**), the bi-antiskyrmion is successfully split into two isolated antiskyrmions. This forced separation is possible since the bi-antiskyrmion is composed of two antiskyrmions with opposite helicities, which rotate in opposite directions. The model is a square element with 40×40 spins. The parameters (in units of $J_1 = 1$) are $J_2 = -0.8$, $J_3 = -1.2$, $K = 0.1$, $H_z = 0.1$, $\alpha = 0.1$, and $j = 8 \times 10^{11}$ A m^{-2}. The simulation on the spin dynamics is carried out by the OOMMF solver integrating the LLG equation including the spin torque.

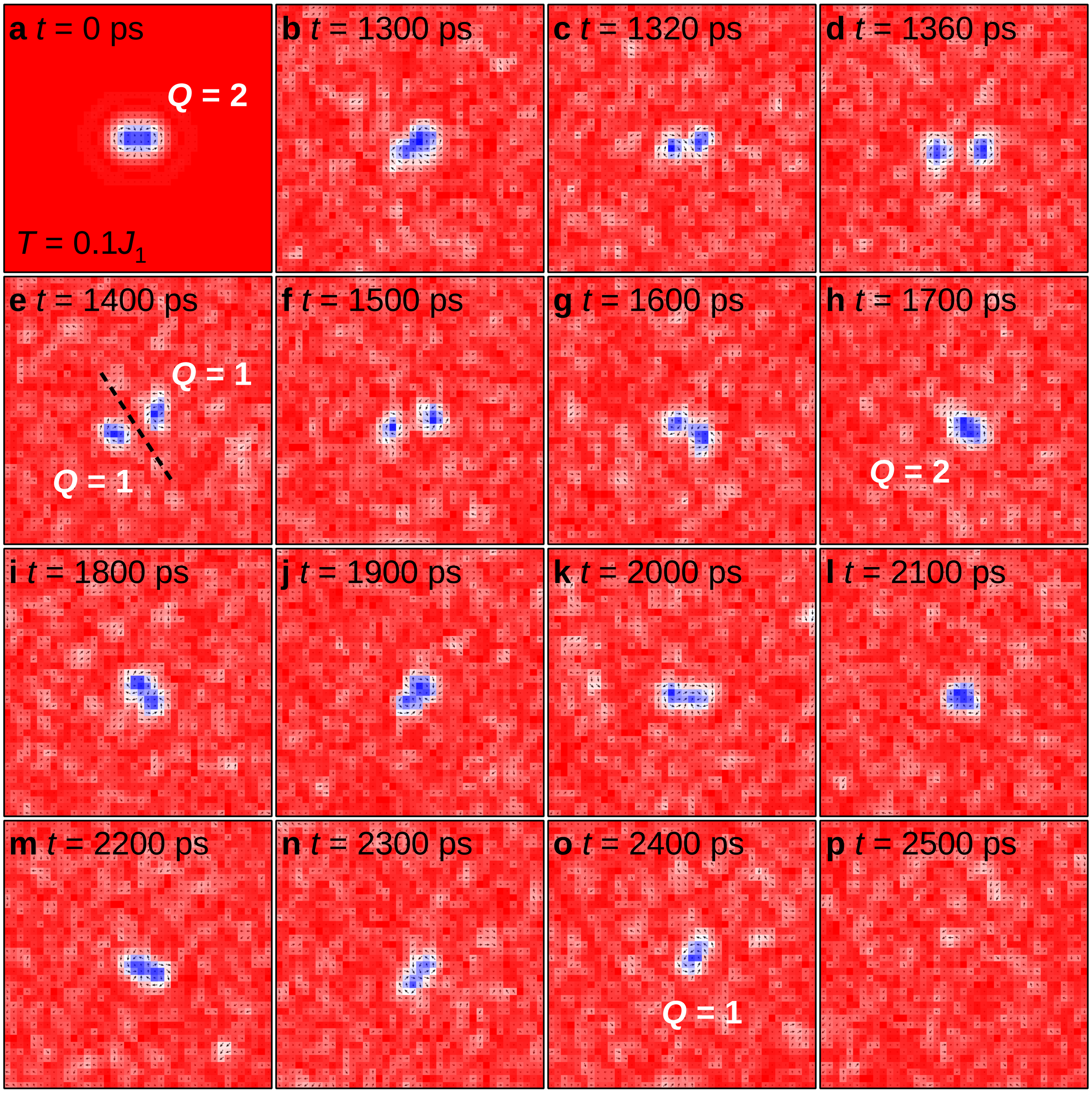

Supplementary Figure 24. **Thermal unbinding and annihilation of a skyrmion bound pair (i.e. a bi-skyrmion) with** $Q = 2$**.** The model is a square element with 40×40 spins. The parameters (in units of $J_1 = 1$) are $J_2 = -0.8$, $J_3 = -1.2$, $K = 0.1$, $H_z = 0.1$, $\alpha = 0.1$, and $T = 0.1$. The simulation is carried out by the OOMMF solver integrating the LLG equation including the spin torque. Under the thermal effect, the bi-skyrmion with $Q = 2$ shows fluctuated behavior including Brown motion and rotation, which could spontaneously separate to form two skyrmions with $Q = 1$, of which could also be re-combined to a bi-skyrmion with $Q = 2$. The bi-skyrmion is ultimately annihilated showing a topological collapse $Q = 2 \rightarrow 1 \rightarrow 0$.

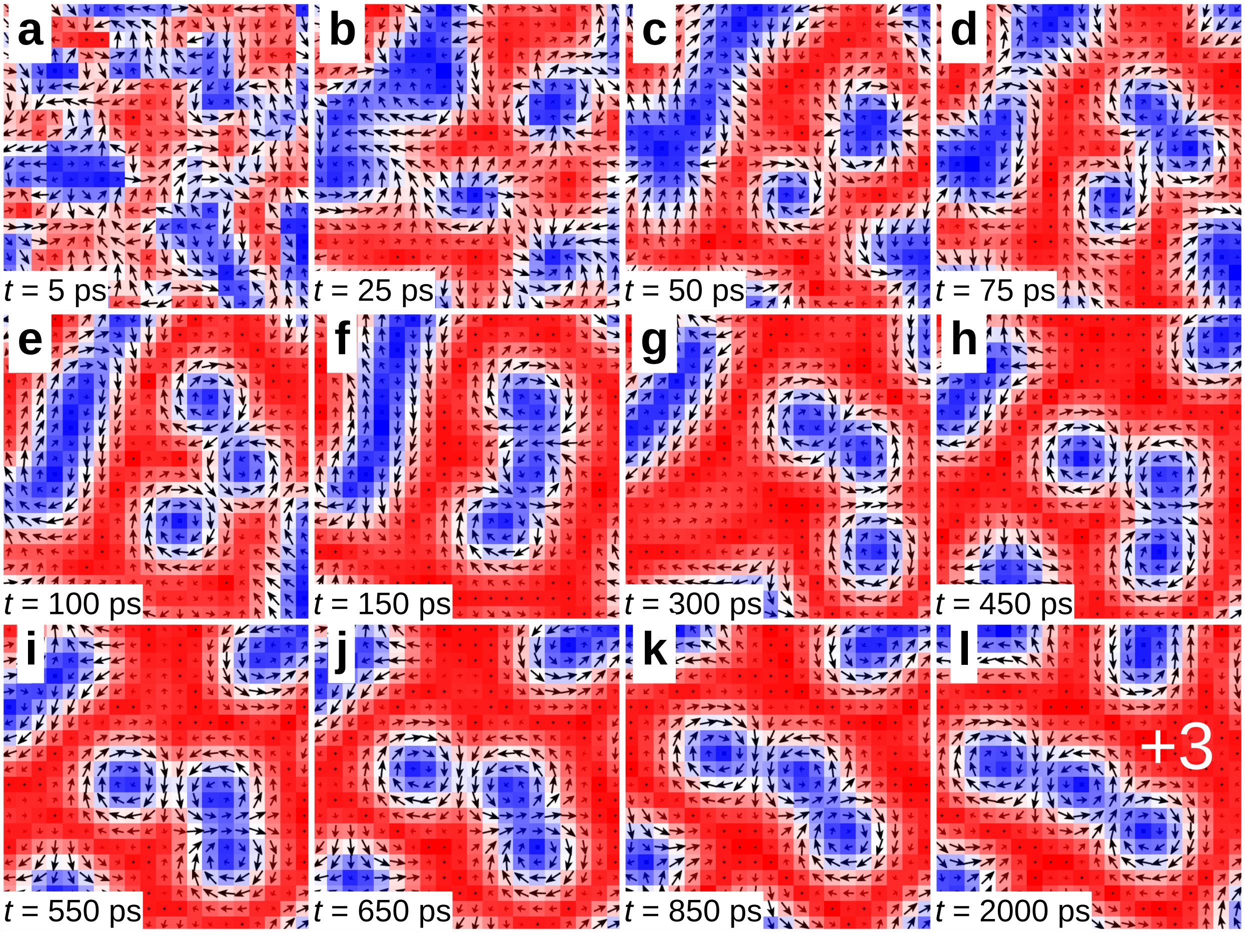

Supplementary Figure 25. **Spontaneous formation of a skyrmion bound state with $Q = 3$ in the relaxation process of a sample with the random initial spin configuration.** A bi-skyrmion ($Q = 2$) is first generated, which further merges with a skyrmion ($Q = 1$) and forms a streptococci-shaped skyrmion bound state with $Q = 3$. The model is a square element with 40×40 spins. The parameters (in units of $J_1 = 1$) are $J_2 = -0.8$, $J_3 = -1.2$, $K = 0.1$, and $H_z = 0.1$. The simulation is carried out by the OOMMF CG minimizer.

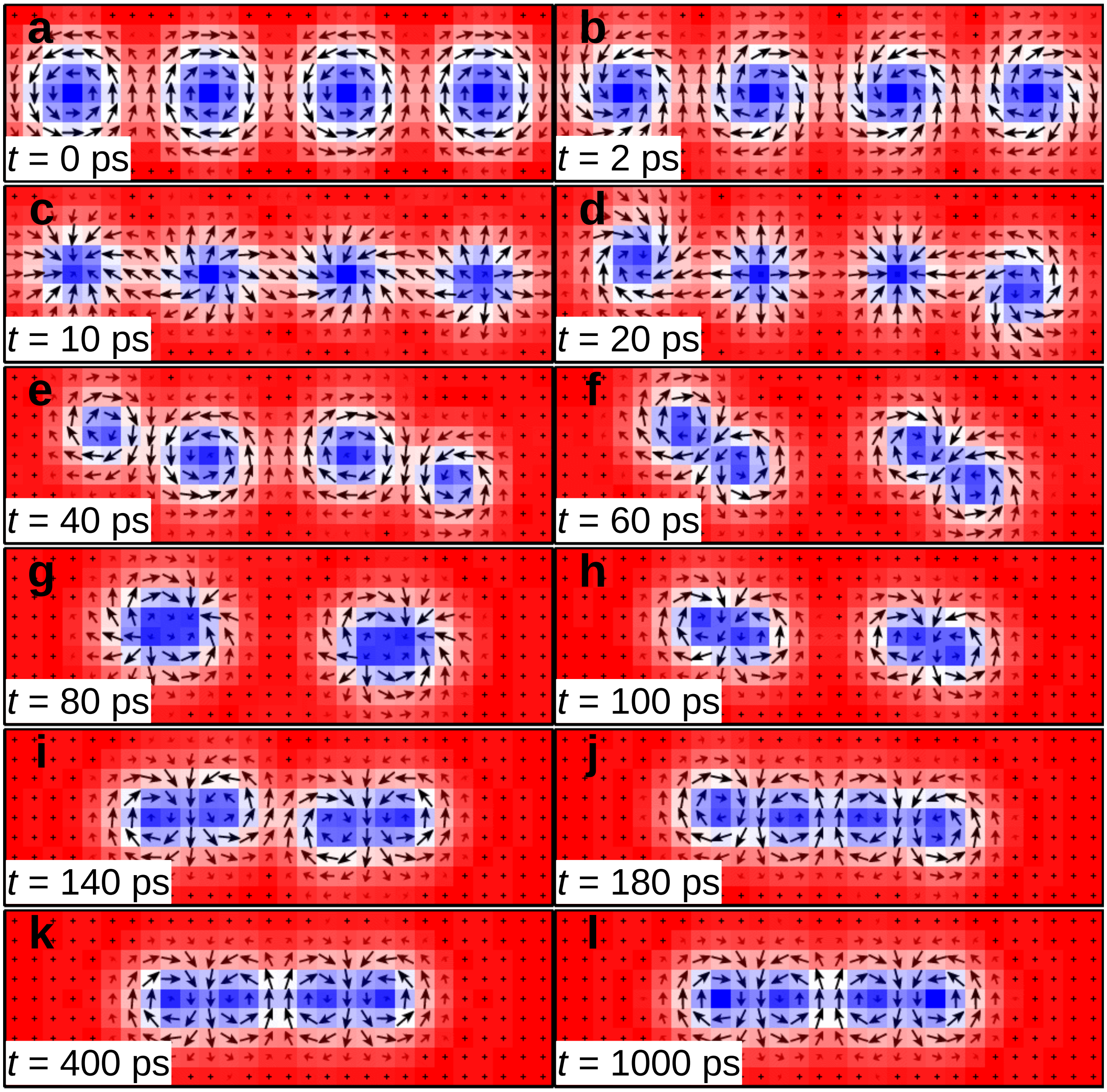

Supplementary Figure 26. **Spontaneous formation of a skyrmion bound state with $Q = 4$.** Four skyrmions are initially placed, from which two bi-skyrmions are generated, and then the two bi-skyrmions continue to merge into a streptococci-shaped skyrmion bound state with $Q = 4$. The model is a square element with 28×9 spins. The parameters (in units of $J_1 = 1$) are $J_2 = -0.8$, $J_3 = -1.2$, $K = 0.1$, $H_z = 0.1$, and $\alpha = 0.1$. The simulation is carried out by the OOMMF solver integrating the LLG equation.

Supplementary Note 1 | Symmetry analysis

A skyrmion is indexed by the skyrmion number Q and the helicity η. We study how the helicity is fixed by minimizing the Hamiltonian. First, we examine the Hamiltonian Equation (1), which is rewritten as

$$H = -\frac{1}{2}\int dqJ\left(\boldsymbol{q}\right)\boldsymbol{m}\left(\boldsymbol{q}\right)\cdot\boldsymbol{m}\left(-\boldsymbol{q}\right) \tag{1}$$

in the momentum space, with

$$\begin{aligned} J\left(\boldsymbol{q}\right) = & -2J_1\left(\cos q_x + \cos q_y\right) \\ & -2J_2\left(\cos\left(q_x - q_y\right) + \cos\left(q_x + q_y\right)\right) \\ & -2J_3\left(\cos 2q_x + \cos 2q_y\right). \end{aligned} \tag{2}$$

The Hamiltonian has the following three symmetries:
1) the inversion symmetry: $q_x \longmapsto -q_x$, $q_y \longmapsto -q_y$;
2) the x-reflection symmetry: $q_x \longmapsto -q_x$, $q_y \longmapsto q_y$;
3) the y-reflection symmetry: $q_x \longmapsto q_x$, $q_y \longmapsto -q_y$.
It is convenient to define the vorticity number Q_v by the winding number of the in-plane spin texture, namely

$$Q_\text{v} = \frac{1}{2\pi}\oint_C d\phi = \frac{1}{2\pi}\left[\phi\right]_{\varphi=0}^{\varphi=2\pi}. \tag{3}$$

We consider the transformation $Q_v \longmapsto -Q_v$ and $\eta \longmapsto -\eta$, under which the skyrmion number is inverted, $Q \longmapsto -Q$. In this symmetry operation, the spins are mapped to

$$m_x \longmapsto m_x, \qquad m_y \longmapsto -m_y, \qquad m_z \longmapsto m_z. \tag{4}$$

The Hamiltonian is invariant with the above transformation. As a result, a skyrmion with Q_v and η and an antiskyrmion with $-Q_v$ and $-\eta$ have the same energy. Moreover, we can show that the energy is independent of η analytically.

The Taylor expansion of the Hamiltonian Supplementary Equation (1) reads

$$\begin{aligned} J\left(\boldsymbol{q}\right) \simeq & -4\left(J_1 + J_2 + J_3\right) + \left(J_1 + 2J_2 + 4J_3\right)\left(q_x^2 + q_y^2\right) \\ & -\left(\frac{J_1}{12} + \frac{J_2}{6} + \frac{4J_3}{3}\right)\left(q_x^4 + q_y^4\right) - J_2 q_x^2 q_y^2. \end{aligned} \tag{5}$$

We chose the values of J_1, J_2 and J_3, which satisfy $J_1 + J_2 + J_3 > 0$ and $\frac{J_1}{12} + \frac{J_2}{6} + \frac{4J_3}{3} < 0$. When $J_1 + J_2 + J_3 < 0$, the skyrmion is not stabilized since q^4 terms do not play an important role. The condition $\frac{J_1}{12} + \frac{J_2}{6} + \frac{4J_3}{3} < 0$ is necessary to cut the expansion with the order of q^4. In the polar coordinate, substituting

$$q_x = \cos\theta\frac{\partial}{\partial r} - \frac{1}{r}\sin\theta\frac{\partial}{\partial\theta}, \qquad q_y = \sin\theta\frac{\partial}{\partial r} + \frac{1}{r}\cos\theta\frac{\partial}{\partial\theta}, \tag{6}$$

the Hamiltonian is rewritten as

$$
\begin{aligned}
H = \int r dr \Bigg[& -8\pi \left(J_1 + J_2 + J_3\right) + 2\pi \left(J_1 + 2J_2 + 4J_3\right) \left(\theta'(r)^2 + \frac{n^2}{r^2}\sin^2\theta(r)\right) \\
& - \left(\frac{J_1}{12} + \frac{J_2}{6} + \frac{4J_3}{3}\right) \left\{ \frac{3\pi}{2}\left(\theta''(r)^2 + \theta'(r)^4 + \frac{n^2+1}{r^2}\theta'(r)^2\right) + \frac{\pi}{r}\theta'(r)\,\theta''(r) \right. \\
& \left. + \frac{n^2\pi\left(4+3n^2\right)}{2r^4}\sin^2\theta(r) + \frac{n^2\pi\sin 2\theta(r)}{2r^2}\left(7\theta'(r) + \theta''(r)\right) + \frac{n^2\pi\cos 2\theta(r)}{2r^2}\theta'(r)^2 \right\} \\
& - J_2 \left\{ \frac{\pi}{4}\left(\theta''(r)^2 + \theta'(r)^4 + \frac{n^2+1}{r^2}\theta'(r)^2\right) - \frac{\pi}{2r}\theta'(r)\,\theta''(r) + \frac{n^2\left(4+n^2\right)}{4r^4}\sin^2\theta(r) \right. \\
& \left. + \frac{n^2\pi\sin 2\theta(r)}{4r^2}\left(5\theta'(r) + \theta''(r)\right) + \frac{3n^2\pi\cos 2\theta(r)}{4r^2}\theta'(r)^2 \right\} \Bigg]. \qquad (7)
\end{aligned}
$$

This is independent of η, which implies that the energy is independent of the helicity. Furthermore, the Hamiltonian is invariant for $n \longmapsto -n$, which implies the energy between the skyrmion and the antiskyrmion is identical irrespective to the helicity η.

Supplementary Note 2 | Thiele equation for the collective coordinate

The collective coordinate ξ_i of a skyrmion is well described by the Thiele equation [1]

$$G_{ij}\dot{\xi}_i + F_j - \mathit{\Gamma}_{ij}\dot{\xi}_i = u\mathit{\Upsilon}_j, \tag{8}$$

with

$$F_i = -\gamma_0 \int \frac{\partial U}{\partial \xi_i} dV, \tag{9}$$

$$\mathit{\Gamma}_{ij} = \alpha \int \frac{\partial \boldsymbol{m}}{\partial \xi_i} \cdot \frac{\partial \boldsymbol{m}}{\partial \xi_j} dV, \tag{10}$$

$$G_{ij} = -\int \boldsymbol{m} \cdot \left(\frac{\partial \boldsymbol{m}}{\partial \xi_i} \times \frac{\partial \boldsymbol{m}}{\partial \xi_j} \right) dV, \tag{11}$$

$$\mathit{\Upsilon}_i = -\boldsymbol{p} \cdot \int \left(\frac{\partial \boldsymbol{m}}{\partial \xi_i} \times \boldsymbol{m} \right) dV, \tag{12}$$

where F_i represents the force with U being the associated potential, $\mathit{\Gamma}_{ij}$ the damping tensor, G_{ij} the gyromagnetic tensor, and $\mathit{\Upsilon}_i$ the current induced torque. Note that u, defined by Equation (4), is proportional to the applied current density j.

We choose the collective coordinate as

$$\boldsymbol{\xi} = (X, Y), \tag{13}$$

from which it follows that

$$\mathit{\Gamma}_{xx} = \mathit{\Gamma}_{yy} = \mathcal{D}\alpha, \quad G_{xy} = G, \quad \dot{\boldsymbol{\xi}} = \boldsymbol{v}, \tag{14}$$

and the Thiele equation is rewritten as

$$\boldsymbol{G} \times \boldsymbol{v} - \mathcal{D}\alpha\boldsymbol{v} = u\boldsymbol{\mathit{\Upsilon}}. \tag{15}$$

By inserting the parametrization [cf. Equation (6)] of $\boldsymbol{m}$ into Supplementary Equation (12), we obtain for skyrmions (+) and antiskyrmions (−) [1]

$$\mathit{\Upsilon}_X^{\pm} = -\pi\mathit{\Upsilon}_0 \cos\eta, \quad \mathit{\Upsilon}_Y^{\pm} = \mp\pi\mathit{\Upsilon}_0 \sin\eta \tag{16}$$

with a constant $\mathit{\Upsilon}_0$ defined by

$$\mathit{\Upsilon}_0^{\pm} = \int_0^{\infty} dr \left[-r\partial_r\theta(r) \mp \frac{1}{2}\sin 2\theta(r) \right]. \tag{17}$$

The mean velocity is given explicitly by solving the Thiele equation as

$$\langle v_x \rangle = \frac{4\pi\sin\eta - \mathcal{D}\alpha\cos\eta}{16\pi^2 + \mathcal{D}^2\alpha^2} u\pi\mathit{\Upsilon}_0, \tag{18}$$

$$\langle v_y \rangle = \mp\frac{4\pi\cos\eta + \mathcal{D}\alpha\sin\eta}{16\pi^2 + \mathcal{D}^2\alpha^2} u\pi\mathit{\Upsilon}_0. \tag{19}$$

The helicity dependence of the velocity is given by this set of equations, which accounts for Fig. 6c of the main text. On the other hand, the helicity-dependent skyrmion Hall angle is given as

$$\Phi_{\text{SkHE}} = \tan^{-1}\left(\mp\frac{4\pi\cos\eta + \mathcal{D}\alpha\sin\eta}{4\pi\sin\eta - \mathcal{D}\alpha\cos\eta} \right). \tag{20}$$

The skyrmion Hall angle is $\Phi_{\text{SkHE}} = 172.6°$ in Fig. 6c of the main text for the stable Bloch skyrmion (e.g., $\eta = \pi/2$), from which we obtain $\mathcal{D} = 16.34$. On the other hand, we find $\mathcal{D} = 15.58$ by the numerical integration of the skyrmion profile based on Supplementary Equations (10) and (14). These values coincide very well.

Supplementary Note 3 | Thiele equation for the helicity

The Thiele equation is also applicable [2] to the helicity η and its conjugate m_z. We now choose the collective coordinate as

$$\boldsymbol{\xi} = (\eta, M_z)\,, \tag{21}$$

from which it follows that

$$\varGamma_{\eta\eta} = \int \left(1 - m_z^2\right) dV, \qquad G_{\eta M_z} = G_{M_z\eta} = 1. \tag{22}$$

The last equation follows from the form of the Berry phase action

$$\int dt(m_z - 1)\dot{\phi}dV = \int dt(M_z - 1)\dot{\eta}dV. \tag{23}$$

The Thiele equations for η and δM_z are given by

$$-\delta\dot{M}_z + \alpha\varGamma_{\eta\eta}\dot{\eta} + u\varUpsilon_\eta = -\frac{\partial U}{\hbar\partial\eta}, \tag{24}$$

$$\dot{\eta} + \alpha\varGamma_{M_zM_z}\dot{M}_z - u\varUpsilon_{M_z} = -K\delta M_z, \tag{25}$$

where $\delta M_z = M_z - M_z^0$ is the deviation of the skyrmion magnetic moment from its value for a free skyrmion. In Supplementary Equation (25), $\varUpsilon_{M_z}$ is independent of the time, and we can neglect the term [2] $\alpha\varGamma_{M_zM_z}\dot{M}$. Taking the time derivative of it, we find $-K\delta\dot{M}_z = \ddot{\eta}$. Substituting this into Supplementary Equation (24), we obtain

$$\frac{1}{K}\ddot{\eta} + \alpha\varGamma_{\eta\eta}\dot{\eta} + u\varUpsilon_\eta = -\frac{dU}{\hbar d\eta}. \tag{26}$$

This is the basic equation.

The DDI induces the potential term U to η. We have numerically shown that the potential term takes the maximum value at the Néel points ($\eta = 0$ and π) and the minimum value at the Bloch points ($\eta = \pi/2$ and $3\pi/2$) as in Fig. 4 of the main text. Such a potential is well approximated by

$$U(\eta) = U_0 \cos 2\eta, \tag{27}$$

with $U_0 \sim 0.02 \times 10^{-20}$ J for the given parameters [cf. Fig. 4a of the main text]. Consequently, the dynamics of the skyrmion motion is equivalent to a forced pendulum problem in the presence of a friction: (i) When the external current is zero (i.e. $u = 0$), we find $\eta = \pi/2$ and $3\pi/2$ as the minimum-energy solution. In this case, from Supplementary Equation (19), a skyrmion undergoes a constant motion. (ii) When the external current is strong enough, the potential term is neglected, and we find $\eta = -(u\pi\varUpsilon_\eta/\alpha\varGamma_{\eta\eta})t$. In this case, from Supplementary Equation (19), a skyrmion undergoes a rotation.

In determining the transition between the two types of motions, we may neglect the Gilbert damping term, and obtain the modified sine-Gordon equation,

$$\frac{1}{K}\ddot{\eta} = 2U_0 \sin 2\eta - u\varUpsilon_\eta = \frac{dV}{\hbar d\eta} \tag{28}$$

with the potential $V(\eta) = U_0/\hbar \cos 2\eta - u\pi\varUpsilon_\eta\eta$. The corresponding Hamiltonian is

$$H = \frac{1}{2K}\dot{\eta}^2 + V(\eta)\,. \tag{29}$$

The condition of the transition is that the sign of $dV/d\eta$ is always the same, which determines the critical value of u as

$$u\Upsilon_\eta = 2U_0/\hbar, \tag{30}$$

yielding Equation (8) with the use of Equation (4).

Supplementary References

[1] Lin, S.-Z. & Hayami, S. Ginzburg-Landau theory for skyrmions in inversion-symmetric magnets with competing interactions. *Phys. Rev. B* **93**, 064430 (2016).

[2] Leonov, A. O. & Mostovoy, M. Edge states and skyrmion dynamics in nanostripes of frustrated magnets. *Nat. Commun.* **8**, 14394 (2017).
