# Supplementary material for: Skyrmion dynamics in a frustrated ferromagnetic film and current-induced helicity locking-unlocking transition
Source: Nat Commun. 2017 Nov 23;8:1717. doi: 10.1038/s41467-017-01785-w (PMC5700181; doi:10.1038/s41467-017-01785-w)
Supplement: Supplementary file 3 — Description of Additional Supplementary Files [file 41467_2017_1785_MOESM3_ESM.pdf]

**Descriptions of Additional Files**

File Name: Supplementary Movie 1
Descriptions: Motion of skyrmions and antiskyrmions with different initial helicity number $\eta$ in a frustrated magnetic film driven by the current in the presence of the DDI.

File Name: Supplementary Movie 2
Descriptions: Motion of skyrmions and antiskyrmions with different initial helicity number $\eta$ in a frustrated magnetic film driven by the current in the absence of the DDI.

File Name: Supplementary Movie 3
Descriptions: Current-induced motions of skyrmions toward opposite directions due to their different helicity numbers. The skyrmion with $\eta = \pi/2$ moves toward the right, while the skyrmion with $\eta = 3\pi/2$ moves toward the left.

File Name: Supplementary Movie 4
Descriptions: Flip of the helicity and the motion direction of a moving skyrmion induced by a strong current pulse.

File Name: Supplementary Movie 5
Descriptions: Spontaneous formation of the bi-skyrmion bound state.

File Name: Supplementary Movie 6
Descriptions: Spontaneous formation of the bi-antiskyrmion bound state.

File Name: Supplementary Movie 7
Descriptions: Forced separation of the bi-skyrmion bound state.

File Name: Supplementary Movie 8
Descriptions: Forced separation of the bi-antiskyrmion bound state.

File Name: Supplementary Movie 9
Descriptions: Pair annihilation of a skyrmion and an antiskyrmion.
